# Next‐Generation Proteolysis‐Targeting Chimeras in Precision Oncology: Multifunctional Designs, Emerging Modalities, and Translational Prospects in Targeted Protein Degradation

**DOI:** 10.1002/ddr.70192

**Published:** 2025-12-08

**Authors:** Mohamed S. Nafie, Mohamed K. Diab, Asmaa S. A. Yassen, Amany M. Elshamy, Mohamed R. El Tohamy, Haytham O. Tawfik, Sherif Ashraf Fahmy

**Affiliations:** ^1^ Department of Chemistry, College of Sciences University of Sharjah Sharjah United Arab Emirates; ^2^ Bioinformatics and Functional Genomics Research Group, Research Institute of Sciences and Engineering (RISE) University of Sharjah Sharjah United Arab Emirates; ^3^ Pest Physiology Department Plant Protection Research Institute, Agricultural Research Center Giza Egypt; ^4^ Department of Medicinal Chemistry, Faculty of Pharmacy Galala University Egypt; ^5^ Pharmaceutical Organic Chemistry Department, Faculty of Pharmacy Suez Canal University Ismailia Egypt; ^6^ Medical Laboratory Science Department, School of Allied Health Sciences Badr University in Cairo (BUC) Badr City Egypt; ^7^ The Faculty of Healthcare Technology Saxony Egypt University for Applied Science & Technology (SEU) Badr City Egypt; ^8^ Department of Pharmaceutical Chemistry, Faculty of Pharmacy Tanta University Tanta Egypt; ^9^ Department of Pharmacy, Institute of Pharmaceutics and Biopharmaceutics Marburg University Marburg Germany

**Keywords:** clinical development, dual‐target degraders, folate‐caged PROTACs, phosphoTACs, photocaged PROTACs, PROTACs, targeted protein degradation, TF‐PROTACs, ubiquitin‐proteasome system

## Abstract

Proteolysis‐targeting chimeras (PROTACs)‐mediated protein degradation has been recently developed as a game‐changing approach in oncology drug development. It represents a paradigm shift from traditional enzyme inhibition to selective protein degradation. PROTACs are different from regular small‐molecule inhibitors because they are heterobifunctional compounds that use the ubiquitin‐proteasome system to breakdown disease‐causing oncogenic proteins. This review discusses the next generation of PROTAC platforms that innovate beyond traditional designs, such as dual‐targeting PROTACS that present a novel mode of action, transcription factor‐targeting PROTACs (TF‐PROTACs), phosphorylation‐dependent PROTACs (PhosphoTACs), and phosphorylation binding chimeras (PhosTACs). In kinase degradation, PROTACs have shown promise in addressing resistance mechanisms and carcinogenic drivers. Despite these advancements, issues with clinical pharmacokinetics, E3 ligase tissue selectivity, and subcellular localization persist. Additionally, the development of bio‐responsive and spatially controlled PROTAC systems, such as photocaged and folate‐caged PROTACs, was fully discussed, which achieves maximal precision in tumor selectivity. Furthermore, ARV‐110 and ARV‐471, as two representative PROTACs, have entered clinical trials, suggesting their potentially broader application. Accordingly, this review provides a critical overview of the design rationales, molecular mechanisms of action, therapeutic utilities, and synthetic issues associated with these innovative modalities, focusing on on their translational implication and pharmacokinetic limitations, as well as potential future clinical applications.

**Abbreviations**
AKT: protein kinase B
ALK: anaplastic lymphoma kinase
AR: androgen receptor
AR‐LBD: androgen receptor ligand‐binding domain
ATM: ataxia telangiectasia mutated kinase
BBB: blood‐brain barrier
BCL2: B‐cell leukemia/lymphoma 2 protein
BCL‐XL: B‐cell lymphoma‐extra large
BCR‐ABL: breakpoint cluster region gene with the Abelson murine leukemia viral oncogene homolog 1 gene
BRD4: bromodomain‐containing protein 4
BTK: Bruton's tyrosine kinase
CDK9: cyclin‐dependent kinase 9
CNS: central nervous system
CPPs: cell‐penetrating peptides
CRBN: cereblon, a protein, plays a role in protein degradation
CRISPR/dCas9: clustered regularly interspaced short palindromic repeat‐associated protein 9
CuAAC: copper‐catalyzed azide‐alkyne cycloaddition
c‐KIT: A receptor tyrosine kinase, that plays a crucial role in cell growth, differentiation, and survival
DBDs: DNA‐binding domains
DC_50_
: half‐maximal degradation concentration
DDR: DNA damage response
DIC/NHS: disseminated intravascular coagulation and N‐hydroxysuccinimide, respectively
DMNB: 4,5‐dimethoxy‐2‐nitrobenzyl
DP‐V‐4: gefitinib‐olaparib‐VHL conjugates
dSTAT3‐1: phosphorylated STAT3
DUBs: deubiquitinases
EDC/HOBt: 1‐ethyl‐3‐(3‐dimethylaminopropyl)carbodiimide hydrochloride and 1‐hydroxybenzotriazole, respectively
EGFR: epidermal growth factor receptor
EML4: echinoderm microtubule‐associated protein‐like 4
ER: estrogen receptor
ERK: extracellular signal‐regulated kinase
F36V: A specific amino acid mutation in a protein, identified by its position (36) and the substitution of valine (V) for another amino acid at that site
FEM1B: Fem‐1 homolog B, a protein plays a role in mediating apoptosis, particularly in colon cancer
FGFR: fibroblast growth factor receptor
FKBP12: FK506‐binding protein 12 that plays a role in cancer cell sensitivity to chemotherapy
FRS2α: fibroblast growth factor receptor substrate 2α
FRα: folate receptor alpha
G1: first stage of tumor grading
G2/M: checkpoint in the cell cycle that prevents cells with damaged DNA from entering mitosis (M phase)
GAS: Gamma‐activated sequence
GCK: glucokinase, an enzyme that plays a crucial role in glucose metabolism
HATU: O‐(7‐azabenzotriazol‐1‐yl)‐N,N,N',N'‐tetramethyluronium hexafluorophosphate, a chemical reagent widely used in peptide synthesis
HER2 and HER2+: human epidermal growth factor receptor 2
HIF‐1α: hypoxia‐inducible factor‐1 alpha
HRMS: high‐resolution mass spectrometry
IC_50_
: half‐maximal inhibitory concentration
IKZF1/3: Ikaros family zinc finger proteins 1 and 3
JAK‐STAT: Janus kinase/signal transducer and activator of transcription signaling pathway
JQ1: BET bromodomain inhibitor
KEAP1: Kelch‐like ECH‐associated protein 1, a gene acts as a tumor suppressor
LBD: ligand‐binding domain of a receptor protein
LNAs: locked nucleic acids
MCL‐1: myeloid cell leukemia sequence 1
mCRPC: metastatic castration‐resistant prostate cancer
MEKK1: mitogen‐activated protein kinase kinase kinase 1
MELK: maternal embryonic leucine zipper kinase
mTOR: mechanistic target of rapamycin
MYC: myelocytomatosis oncogene
NF‐κB: nuclear factor kappa B
NSCLC: nonsmall cell lung cancer
p53: tumor protein 53
p65: RelA, a protein subunit of the NF‐κB transcription factor complex
PARP: poly (ADP‐ribose) polymerase
PEG: polyethylene glycol
PEG_3_
: triethylene glycol
PI3Kα: phosphatidylinositol‐3 kinase subunit alpha
PLK1: polo‐like kinase 1
POI: protein of interest
PPIs: protein–protein interactions
PROTACs: proteolysis‐targeting chimeras
PSA: prostate‐specific antigen
RNF114: RING finger protein 114
RTKs: receptor tyrosine kinases
SAR: structure–activity relationship
SD‐36: STAT3 PROTAC degrader
Ser2: serine 2, which is a specific amino acid residue within a protein
Ser5: serine 5, which is a specific amino acid residue within a protein
SH2: Src homology 2
SI‐109: potent STAT3 inhibitor with demonstrated antitumor activity
SNS‐032: CDK inhibitor (cyclin‐dependent kinase inhibitor) with potential anticancer activity
SPPS: solid‐phase peptide synthesis
STAT3: signal transducer and activator of transcription 3
TFs: transcription factors
TPD: targeted protein degradation
UPS: ubiquitin proteasome system
VH032: VHL ligand
VHL: von Hippel‐Lindau
γH2AX: Gamma‐H2AX refers to a phosphorylated form of the histone protein H2AX, a biomarker in cancer research and treatment to monitor DNA damage

## 1 Introduction

**Figure 1:**
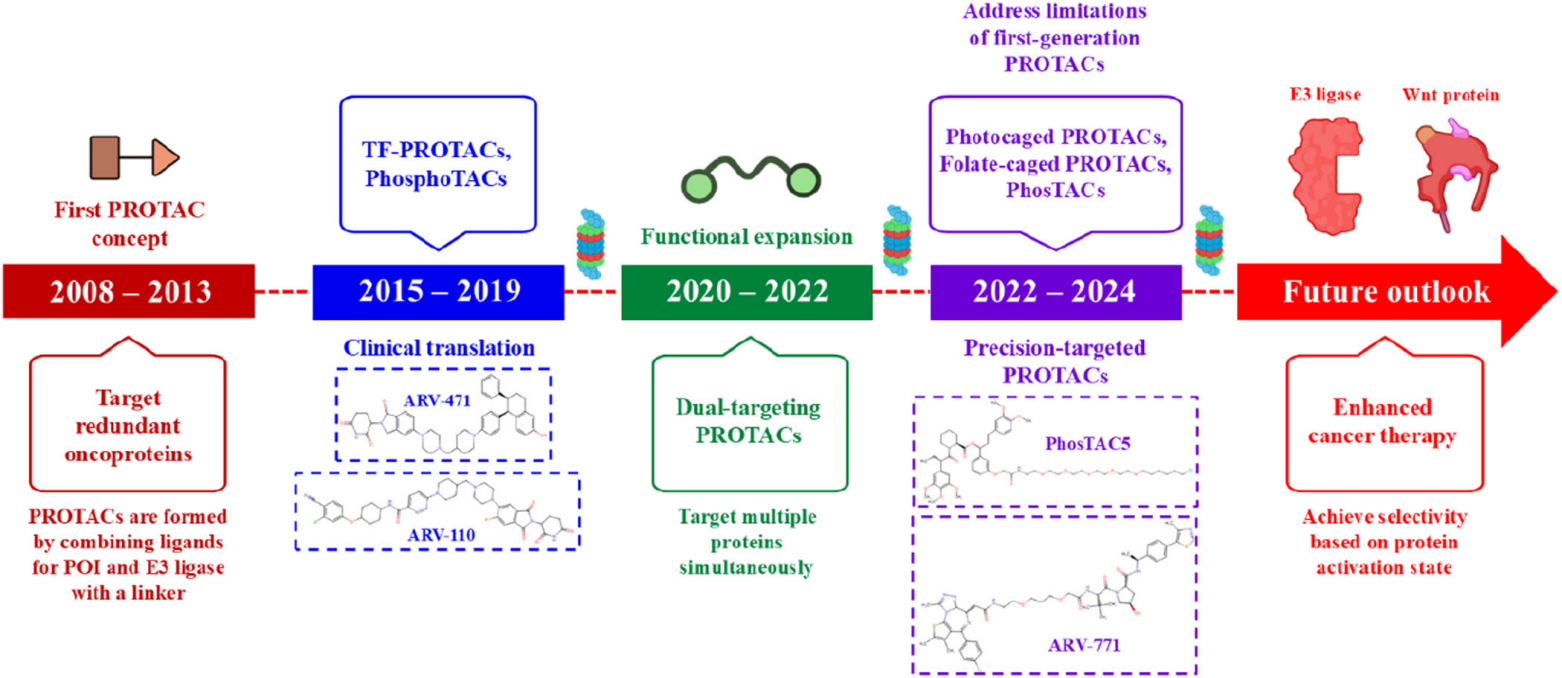
Key milestones in the evolution of PROTACs and targeted protein degradation.

Targeted protein degradation (TPD) has emerged as an exciting new therapeutic approach, particularly in the field of oncology, where it overcomes the common limitations associated with traditional small‐molecule inhibitors (Békés et al. 2022). An event‐driven activity of proteolysis targeting chimeras (PROTACs) targeting disease‐relevant proteins through ubiquitin proteasome system (UPS)‐specific degradation has drawn considerable interest in the domain of targeted protein degradation (TPD) strategies (Kamaraj et al. 2024). PROTACs act catalytically and can eliminate both enzymatic and nonenzymatic targets, resulting in sustained pharmacological activity and the potential to overcome resistance mechanisms (Figure 1). In contrast, traditional occupancy‐based inhibitors require continuous active site engagement (Liu, Hu et al. 2022).

**Figure 2:**
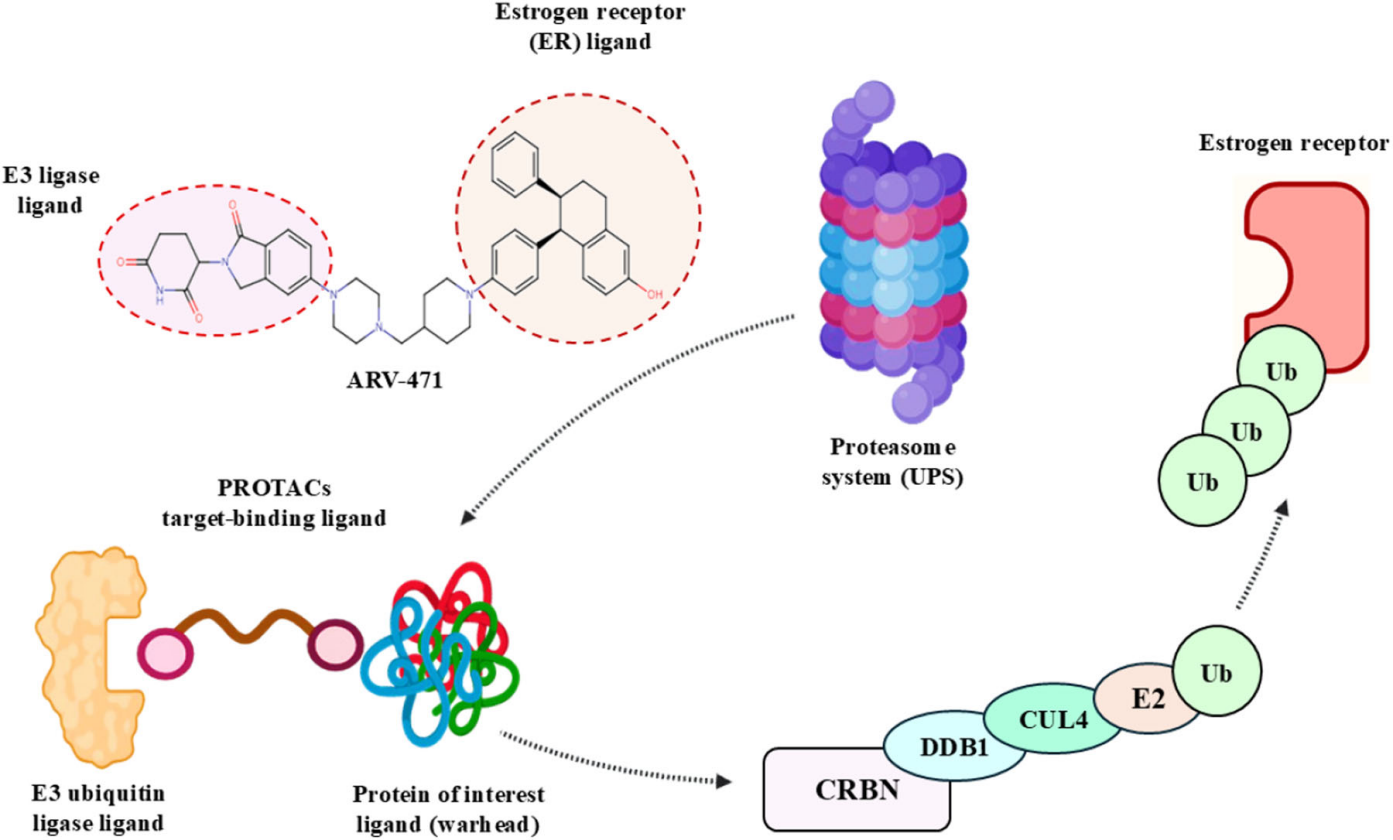
Mechanism of Vepdegestrant (ARV‐471) dual‐PROTACs degrader targeting estrogen receptor (ER) ligand in breast cancer.

A ligand that binds the protein of interest (POI), another ligand that attracts an E3 ubiquitin ligase (like Von Hippel‐Lindau [VHL] or Cereblon [CRBN]), and a flexible linker that permits the formation of a ternary complex to initiate ubiquitination and subsequent degradation of the POI comprise the three primary components of classical PROTACs, which are heterobifunctional molecules (Pichlak et al. 2023). As exemplified by the clinical candidates ARV‐110 and ARV‐471, respectively, it has been possible to develop PROTACs against targets previously considered undruggable, including androgen receptor (AR) in prostate cancer and estrogen receptor (ER) in breast cancer, as illustrated in (Figure 2) (Dogheim and Amralla 2023; Snyder et al. 2025).

Although first‐generation PROTACs still have several limitations, such as an ineffective degradation capacity for structurally intractable proteins (e.g., transcription factors [TFs]), an inability to differentiate the active signaling protein from its inactive form, and the possibility of compensation by parallel survival pathways (Guedeney et al. 2023). Next‐generation PROTAC technologies, which aim to enhance precision and expand the range of degradable targets, have been developed in response to these challenges (Wang, Zhang, Chen et al. 2024).

In this study, our attention is directed to new features of the PROTAC design that promote the development of more complex approaches to cancer treatment. Dual‐targeted PROTACs, targeting functionally redundant or compensatory oncoproteins, are also highlighted (Mancarella et al. 2023). These are TF‐PROTACs, which recruit TFs to DNA oligonucleotides for degradation (Liu, Chen, Kaniskan et al. 2021). Activation state selectivity is also accomplished with phosphorylation‐dependent interactions by the PhosphoTACs. When combined, these emerging approaches not only extend the druggable target space but also offer insights into long‐standing challenges in cancer therapy (Kubryń et al. 2025). Overall, the current limitations in E3 ligase selectivity, pharmacokinetics, and route of administration, along with proposed future strategies, were discussed to advance PROTAC‐directed therapy closer to clinical translation.

## 2 Mechanism of Action of Classical Proteolysis‐Targeting Chimeras (PROTACs)

Protein degradation by the endogenous UPS is an emerging approach in drug discovery and development, known as PROTACs, which can induce the selective degradation of endogenous intracellular proteins (Kamaraj et al. 2024). The mechanism of action of PROTACs is driven by a catalytic cycle, which selects the physical destruction of the target protein and thus potentially leads to extensive pharmacodynamic effects after the molecule is removed from the system. This is in contrast to canonical inhibitors, which inhibit protein function in a direct and sustained manner (Ou et al. 2025).

Classical PROTACs are structurally unique small molecules that consist of a ligand for binding to a protein of interest (POI), a ligand for binding to an E3 ubiquitin ligase (usually CRBN or VHL), and a linker designed to position both these ligands in a way that will permit the formation of a ternary complex between PROTAC, POI, and an E3 ligase (Diehl and Ciulli 2022).

Ubiquitin moieties are then transferred from the E2 enzyme to lysine residues on the target protein by the E3 ligase once the ternary complex is formed successfully, leading to recognition of the protein by the 26S proteasome and its subsequent degradation (Sosič et al. 2022). Comparing this event‐based approach to occupancy‐based inhibition offers several advantages: Due to their catalytic action, individual PROTACs can potentially degrade many protein molecules without the need for sustained exposure (Chen et al. 2022). They can target regulatory proteins without active sites, as well as nonenzymatic proteins, such as scaffolds. Since the protein is eliminated rather than inhibited, they offer a way to overcome drug resistance caused by target overexpression or mutations that decrease inhibitor binding (He et al. 2022).

The formation and stability of the ternary complex are key determinants that define the degradation efficiency. This interaction is often associated with positive cooperativity, where the binding of one partner enhances the affinity of the other, rather than their binding being an additive combination of the affinities of the separated binary components (Tang et al. 2023). The higher processivity of ubiquitination and the increased potency for degradation reflect the successful formation of the productive ternary complex, as observed in many studies. In addition, the precise structure and productive orientation of the E3 ligase concerning the target protein are significantly aided by the composition, length, and flexibility of the linker (Hu and Crews 2022).

The selection of E3 ligase is another critical factor for the development of classical PROTACs. Even though the human genome contains over 600 E3 ligases, VHL and CRBN are monitoring which E3 ligases are utilized most by cancers, recognizing that the reasons are their characterized ligands, expression profiles, and target range compatibility (Diehl and Ciulli 2022). The subcellular localization of E3 ligases and tissue‐specific E3 ligase expression may, however, impact the in vivo efficacy of PROTACs and thereby their therapeutic window (Kannt and Đikić 2021).

The drug discovery space of modulating protein turnover through degradation can be best illustrated by classical PROTACs (Li, Hogenhout et al. 2025). While early prototypes have demonstrated efficacy in preclinical and clinical studies, off‐target effects, limited oral bioavailability, and poor cellular permeability underscore the need for further optimization (Syahputra et al. 2025). Nonetheless, the basic principles of PROTAC‐induced ubiquitination and degradation remain as a scaffold to be elaborated on to develop ever more sophisticated degraders of the type that could be used to treat hitherto untreatable diseases (Békés et al. 2022).

A further advantage of PROTACs is that they can convert weak or noninhibitory binders into strong degraders (Wang, Zhang, Yu et al. 2024). Since PROTACs operate through a ternary and event‐driven mechanism, their function is not only independent of binding affinity but can also be orthogonal to it; what truly matters is whether an E3 trigger group enables stable proximity between the E3 ligase and the protein of interest (POI) (Vikal et al. 2025; Saluja et al. 2024). This procedure allows for the development of fragments, allosteric binders, or compounds previously set aside due to poor inhibition profiles. For instance, low‐potency BET bromodomain inhibitors have been converted to potent BRD4 degraders with the use of PROTAC scaffolding (Burslem et al. 2018). In addition, weak kinase binders and metabolically labile inhibitors were salvaged by PROTAC conversion for BTK and CDK9 targets (Dong et al. 2024; Li, Yao et al. 2022). This pharmacology change broadens the range of potential druggable targets and enhances therapeutic selectivity, especially for resistant or mutation‐driven oncoproteins.

## 3 Classification of Next‐Generation PROTACs

In addition to classic monovalent degraders, new subclasses have been developed with the expansion of the PROTAC field. Initially, investigators developed selective PROTACs to address problems such as the eligibility of targets, redundancy of proteins, and the inaccessibility of undruggable targets, which became more pronounced over time (Liu, Hu et al. 2022). A functional classification system has been proposed that classifies the next generation of PROTACs based on their structure and their molecular modes of action (Wang, Zhang, Chen et al. 2024).

## 4 Dual‐Target PROTACs: Overcoming Compensatory Resistance

Dual‐target PROTACs represent a cutting‐edge therapeutic approach that simultaneously breaks down two functionally related oncoproteins within a single molecular structure (Mahajan et al. 2025). Adaptive or compensatory resistance represents a severe limitation of monotherapeutic strategies, where modulation of one target results in the induction or increased expression of parallel survival pathways (Burke et al. 2022). This design overcomes this issue. For instance, suppression of EGFR can stimulate PARP‐mediated DNA repair in EGFR‐mutant squamous cell lung cancer, leading to increased tumor cell survival. Dual‐targeted PROTACs lead to the degradation of both proteins, hopefully preventing this escape (Zheng et al. 2021).

**Figure 3:**
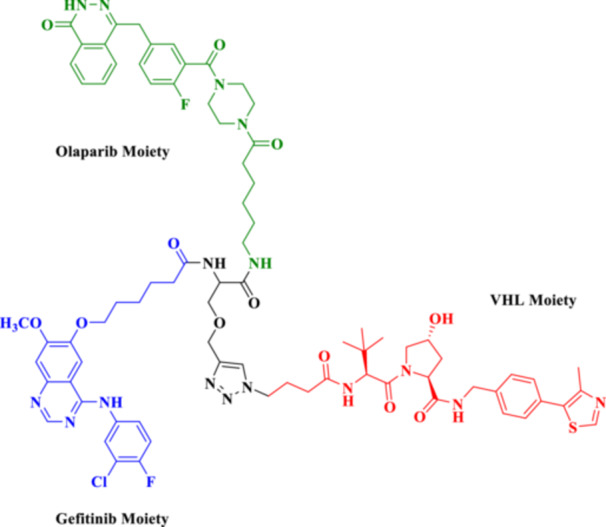
Chemical structure of **DP‐V‐4** as a dual‐target PROTAC.

This bifunctional agent acts as a backbone for two ligands that are capable of targeting two proteins and, at the same time, recruiting an E3 ubiquitin ligase (VHL, CRBN, designed through linkers with specific geometry) (Diehl and Ciulli 2022; Cao et al. 2022). This includes compounds, for example, DP‐V‐4, a chimeric molecule composed of a VHL‐recruiting component (Figure 3), a PARP inhibitor (olaparib), and an epidermal growth factor receptor (EGFR) inhibitor (gefitinib). Through the proximity between the EGFR and PARP and the ligase, DP‐V‐4 creates a ternary complex that leads to the simultaneous ubiquitination and degradation of the two proteins (Yan et al. 2022).

This combinatorial mode of degradation has several therapeutic advantages over classic combination therapies (Liang et al. 2024). Kinetics of synergistic degradation, which ensures that both targets are rapidly depleted and that compensatory signaling does not occur (Riching et al. 2022). The catalytic efficiency allows the use of minimal amounts and less systemic toxicity. Fewer opportunities for resistance as the probability for two targets to mutate or escape degradation concurrently is small (Burke et al. 2022). Pharmacodynamic incoherence, which enhances the efficacy of therapy by synchronizing the two oncoproteins for degradation (Kelm et al. 2023).

DP‐V‐4 showed superior activity compared with that of single‐agent degraders in preclinical models, where it displayed over 70% tumor growth inhibition, particularly in osimertinib‐resistant EGFR‐mutant NSCLC, demonstrating the potential of this strategy to deplete redundant oncogenic pathways (Maity et al. 2023). Despite these promising results, challenges remain in optimizing linker flexibility and length to ensure balanced ternary complex formation (Pichlak et al. 2023). In addition, prevention of the hook effect, characterized by an excessive amount of PROTACs that decrease the best possible complex assembly at higher concentrations, and enhancement of selectivity by the induction of complexes that target larger combinations (e.g., BRD4/BCL2 in leukemia) and even it with tissue‐specific E3 ligases (Moreau et al. 2020; Nieto‐Jiménez et al. 2022).

The development of dual‐targeted PROTACs opens the door to next‐generation, systems‐based cancer treatments and strategies to combat treatment resistance (Békés et al. 2022). Given the requirement to pair two unrelated selective ligands for different protein targets with an E3 ligase‐recruiting moiety within a single molecular structure, the synthesis of a dual protein target PROTAC is particularly challenging (He et al. 2022). These PROTACs typically consist of three functional elements: an E3 ligase‐recruiting moiety (such as VH032 for VHL), a ligand for target protein B (such as PARP), and a ligand for target protein A (such as EGFR). A central linker network links these to allow for coordinated binding and degradation (Diehl and Ciulli 2022; Li, Pu et al. 2022).

In the first step, each ligand is modified chemically to introduce a functional group such as amine, carboxylic acid, or azide at a remote position from the ligand's active binding site (Bricelj et al. 2021). These modifications should enable site‐specific conjugation while maintaining the binding affinity. Molecular docking or structure–activity relationship (SAR) studies are commonly employed to aid in the rational selection of sites for alteration (Li, Zhang et al. 2025). After the functionalized ligands are synthesized, the core linker structure is determined to connect them (Weerakoon et al. 2022). Branched or tripodal linkers (e.g., tris(hydroxymethyl)aminomethane scaffolds) are commonly selected for dual‐target PROTACs, enabling the simultaneous tethering of three groups (He et al. 2022). Allowing linker design to ensure spatial flexibility and optimal positioning of the ligand is essential for the two targets and the E3 ligase to engage to form a ternary complex. For linker length and solubility control, rigid alkyl/triazole‐containing core molecules or polyethylene glycol (PEG)‐based linkers are commonly employed (Kumar and Sobhia 2024).

For carboxylic acid‐containing precursors, chemical coupling is often carried out using amide bond production techniques with activating agents such as DIC/NHS, EDC/HOBt, or HATU (Bakulina et al. 2022). In certain situations, high‐yield conjugation between intermediates functionalized with azides and alkynes is achieved through click chemistry (e.g., CuAAC) (Yang et al. 2024). The employment of reactive functional groups sensitive to such conditions also often requires protection/deprotection protocols. One of the synthetic challenges is to handle the high molecular weight (often > 1000 Da) and lipophilicity of the final molecule, which may negatively impact the drug‐like properties of the molecule (Syahputra et al. 2025). Moreover, to avoid the hook effect, in which high PROTAC concentrations hinder the formation of ternary complexes, the linker length and shape must be optimized (Weerakoon et al. 2022).

Gefitinib and olaparib derivatives were conjugated with a VHL ligand using a tripodal linker to engender DP‐V‐4, a dual degrader of EGFR/PARP (Zheng et al. 2021). It has been demonstrated that this molecule forms stable ternary complexes that facilitate the co‐degradation and ubiquitination of both targets (Zhao 2024). The tumorigenesis in osimertinib‐resistant NSCLC was significantly inhibited by DP‐V‐4, indicating that this dual‐target strategy is promising for further clinical applications (Cordani et al. 2024).

## 5 TF‐PROTACs: Targeting the “Undruggable” TFs

TFs, despite their crucial role as important regulators of gene expression, have been generally viewed as undruggable due to their specific structural characteristics, including flat and flexible surfaces and the absence of well‐defined binding sites (Lin et al. 2025). In cancer, only a few TFs can be effectively targeted using conventional small molecule inhibitors, which has limited the possibilities for therapy (Zhang et al. 2025). In recent years, PROTAC technology has given people an aspirational reason to hope. Darzynkiewicz's group has recently successfully employed TF‐targeting PROTACs (TF‐PROTACs) that, by recruiting E3 ubiquitin ligases to TFs, lead to ubiquitination and proteasomal degradation of TFs (Liu, Chen, Kaniskan et al. 2021).

Currently, there are two major design strategies for TF‐PROTACs: ligand‐based and oligonucleotide‐based. The ligand‐based approach utilizes small molecules that interact with protein partners or cofactors of the TF, thereby modulating its activity (Li, Song et al. 2022). Downregulation of MYC through the indirect mechanism of BRD4 inhibition is one of the most common cases. In this process, thalidomide, VH032, an E3 ligase ligand, is linked to JQ1, a small‐molecule inhibitor of BRD4, to produce a functional PROTAC. This strategy disrupts the MYC expression by the MYC–BRD4 interaction (Mancarella et al. 2023).

**Figure 4:**
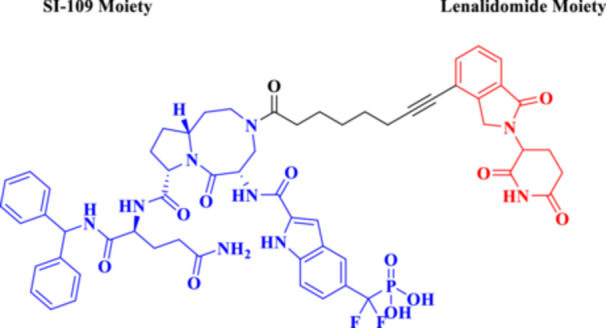
The chemical structure of **SD‐36** as TF‐PROTAC.

More recently, research has focused on the development of PROTACs, such as SD‐36 (Figure 4), which target STAT3, a TF overexpressed in many types of cancer (Zhou et al. 2019). Consistently, dSTAT3‐1, as well as SI‐109 and SD‐36, are small‐molecule ligands targeting the SH2 domain of STAT3 (Bai et al. 2019). It is bound to a CRBN‐binding ligand such as pomalidomide by a proper spacer. SD‐36 exhibited potent STAT3 degradation in leukemic cell lines while showing minimal effects on other members of the STAT family (He et al. 2022). A single intravenous dose of SD‐36 led to marked tumor regression and sustained STAT3 inhibition in animal models, making it an attractive model for ligand‐based TF‐PROTACs (Jin et al. 2022).

Other ligand‐based TF‐PROTACs have also been developed based on their interactions with druggable partners, which could degrade coactivators, such as p65 (NF‐κB) or HIF‐1α (Li, Song, et al. 2022). The classical protocols for PROTAC synthesis, including the introduction of functional groups into small molecules (e.g., alkyne, amine) and the conjugation to E3 ligase ligands by coupling reactions, such as click chemistry or amide bond formation, are frequently used to achieve the chemical synthesis of ligand‐based TF‐PROTACs (Bricelj et al. 2021).

The oligonucleotide‐based strategy offers a faster alternative to drug design points as it targets the DNA‐binding domains (DBDs) of TFs (Zhang et al. 2025). In this approach, synthetic oligonucleotides are designed and created to mimic native TF binding sequences, such as those found in promoter or enhancer elements (Liu, Chen, Kaniskan et al. 2021). To resist degradation by nucleases, these oligos are chemically modified, featuring, for example, 2′‐O‐methylated groups, a phosphorothioate backbone, or locked nucleic acids (LNAs) (Mancarella et al. 2023). A well‐documented example is the conjugation of a phosphorothioate‐modified oligonucleotide under the GAS (gamma‐activated sequence) promoter with a VHL ligand (Diehl and Ciulli 2022). STAT3 is the target of this TF‐PROTAC, which binds to STAT3 via its DBD and recruits VHL ligase for proteasomal degradation (Li, Wang et al. 2025). This oligo‐PROTAC also significantly inhibited the growth and metastasis of triple‐negative breast cancer models, and decreased STAT3 levels by more than 80% (Jin et al. 2022).

**Figure 5:**
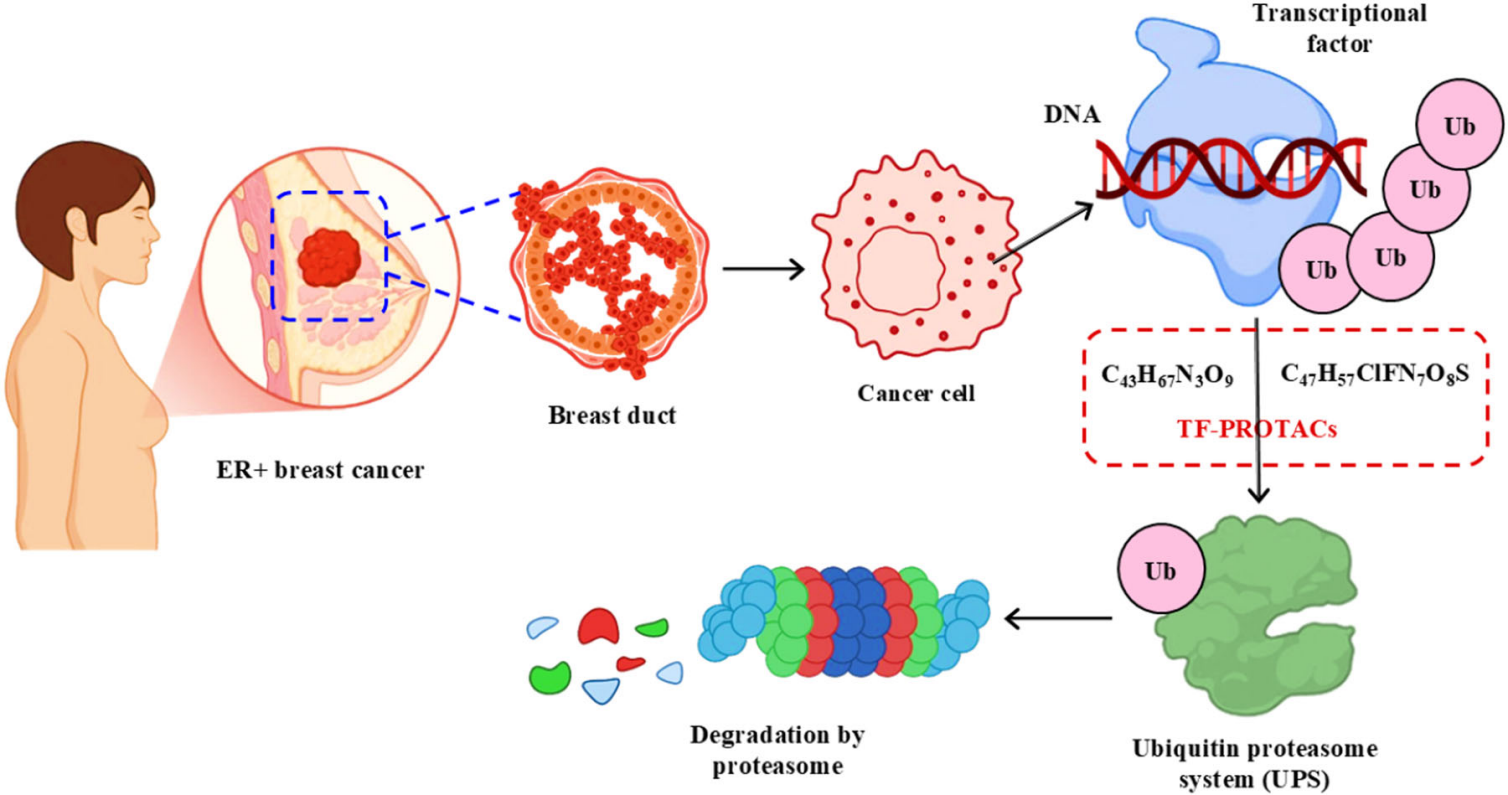
TF‐PROTACs targeting estrogen receptor (ER) in breast cancer.

The oligo‐based PROTACs consist of three structural blocks: a linker possessing reactive handles (as in DBCO‐azide), a double‐stranded DNA decoy that exhibits high binding affinity for the TF DBD, and a small‐molecule ligand for the E3 ligase (such as VH032 for VHL) (Bricelj et al. 2021). The last complex is formed through bioconjugation strategies, such as maleimide‐thiol coupling or azide‐alkyne cycloaddition (also known as click chemistry) (Yang et al. 2024). The STAT3‐D‐PROTAC is a model case in point, where a PEG linker is used to tether the decoy oligonucleotide with a VHL ligand, enabling the formation of ternary complexes and efficient STAT3 ubiquitination and degradation, as shown in (Figure 5) (Li, Wang et al. 2025; Hall et al. 2024).

There are still many challenges that need to be overcome in the development of TF‐PROTACs. Druggable cofactors are also a limiting factor for ligand‐based approaches, while oligonucleotide‐based ones will need to circumvent issues related to intracellular targeting, endosomal escape, and delivery (Syahputra et al. 2025). Novel technologies, including cell‐penetrating peptides (CPPs) for enhanced penetration, endosomal escape enhancers for improved escape, and CRISPR/dCas9 for targeted localization, have been actively investigated to overcome these challenges (Kanbar et al. 2024). All things considered, TF‐PROTACs provide new avenues for the challenging pursuit of TF modulation. Their development using either ligand‐ or oligonucleotide‐based methodologies is an innovative breakthrough in the field of next‐generation drug discovery and targeted protein degradation (Liu, Chen, Kaniskan et al. 2021).

## 6 PhosphoTACs: A Signal‐Dependent Degradation Strategy

A new type of targeted protein degraders, phosphorylation‐dependent PROTACs (phosphorylation‐TACs), exploits phosphorylation to trigger the selective degradation of proteins in a context‐sensitive manner (Hollingsworth et al. 2023). PhosphoTACs are predesigned to degrade proteins in a phosphorylation‐dependent manner, unlike original PROTACs, which work independently of the activity level of their targetable protein. This can lead to a higher level of selectivity and fewer off‐target effects in the case of adaptor proteins or transient signaling molecules that do not contain an enduring, druggable pocket or enzymatic activity (Xiao et al. 2022).

A phospho‐recognition element, primarily a phosphomimetic peptide or small‐molecule analogue, comprised a core of a phosphoTAC (Kubryń et al. 2025). It specifically binds to the phosphorylated form of the target protein. A flexible linker extends from this motif to the E3 ligase ligand, for example, pomalidomide (for cereblon, CRBN) or VH032 (for Von Hippel‐Lindau, VHL) (Diehl and Ciulli 2022). Computational modeling can be used to tune the linker to optimize the shape of the ternary complex formed between the E3‐ligase, the phospho‐specific recognition motif, and the target (Miller et al. 2023). PhosphoTACs can be prepared by solid‐phase peptide synthesis (SPPS), which may also include nonhydrolyzable phospho‐analogs, such as (phosphonodifluoromethyl)phenylalanines or difluorophosphonates, for enhanced cellular uptake and metabolic stability (Makukhin and Ciulli 2021).

Downregulation of FRS2α, a downstream adaptor protein of the FGFR (fibroblast growth factor receptor) pathway, represents an example of one of the first phosphoTACs. In this procedure, a VHL ligand was conjugated to a phosphorylated‐mimic peptide synthesized using the phosphorylated sequence of FRS2α as a template and incorporating an aminohexanoic acid linker (Kong et al. 2024). FRS2α is phosphorylated upon FGFR activation, enabling the phosphoTAC to bind preferentially and to recruit the E3 ligase machinery (Ma et al. 2023). The GRB2–SOS–RAS–MAPK signaling pathway is blocked by the ubiquitination and proteolysis of FRS2α. Under conditions of active FGFR, this phosphoTAC displayed signal‐dependent activity, resulting in efficient destruction (Wang, Liu et al. 2024).

**Figure 6:**
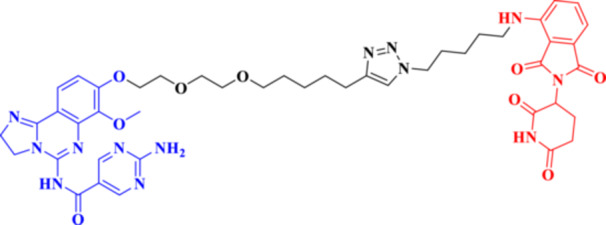
The chemical structure of **ZM‐PI05** as PhosphoTAC.

Use of a phosphoTAC that degrades PI3Kα (ZM‐PI05) (Figure 6), an essential lipid kinase in the pathway of the PI3K–AKT, which is more significant in HER2‐amplified breast cancers, can be similarly significant (Zhong et al. 2024). The PI3Kα pocket is accessible only when the recognition moiety of this phosphoTAC docks to PI3Kα upon downstream phosphorylation by activated receptor tyrosine kinases (RTKs), like HER2 (Zhang et al. 2020). When phosphorylated, the phosphoTAC utilizes a VHL ligand to recruit PI3Kα to the E3 ligase complex, resulting in the selective degradation of the active pool of PI3Kα (Wang et al. 2022). Notably, in mouse models, this degradation method reduced tumor development. It induced a more prolonged inhibition of AKT signaling while minimizing damage in normal tissues, outperforming the conventional catalytic inhibitor Alpelisib (Zhang, Zhang et al. 2024).

Because phosphoTACs are designed to induce degradation of a phosphorylated protein kinase independently of the kinase having supplied the phosphorylation (Yu et al. 2021). Proteins from different pathways can be treated more flexibly. This approach is limited by dependence on phosphorylation pathways and peptide‐based motif degradation, as well as the difficulty of achieving effective nuclear localization and cell penetration (He et al. 2025). Recent progress is focused on the introduction of chemically stable nonpeptidic phosphobinders and the extension of the target repertoire to pathways such as Hippo and JAK‐STAT for which phosphorylation dynamics are regulated (Mancarella et al. 2023).

**Figure 7:**
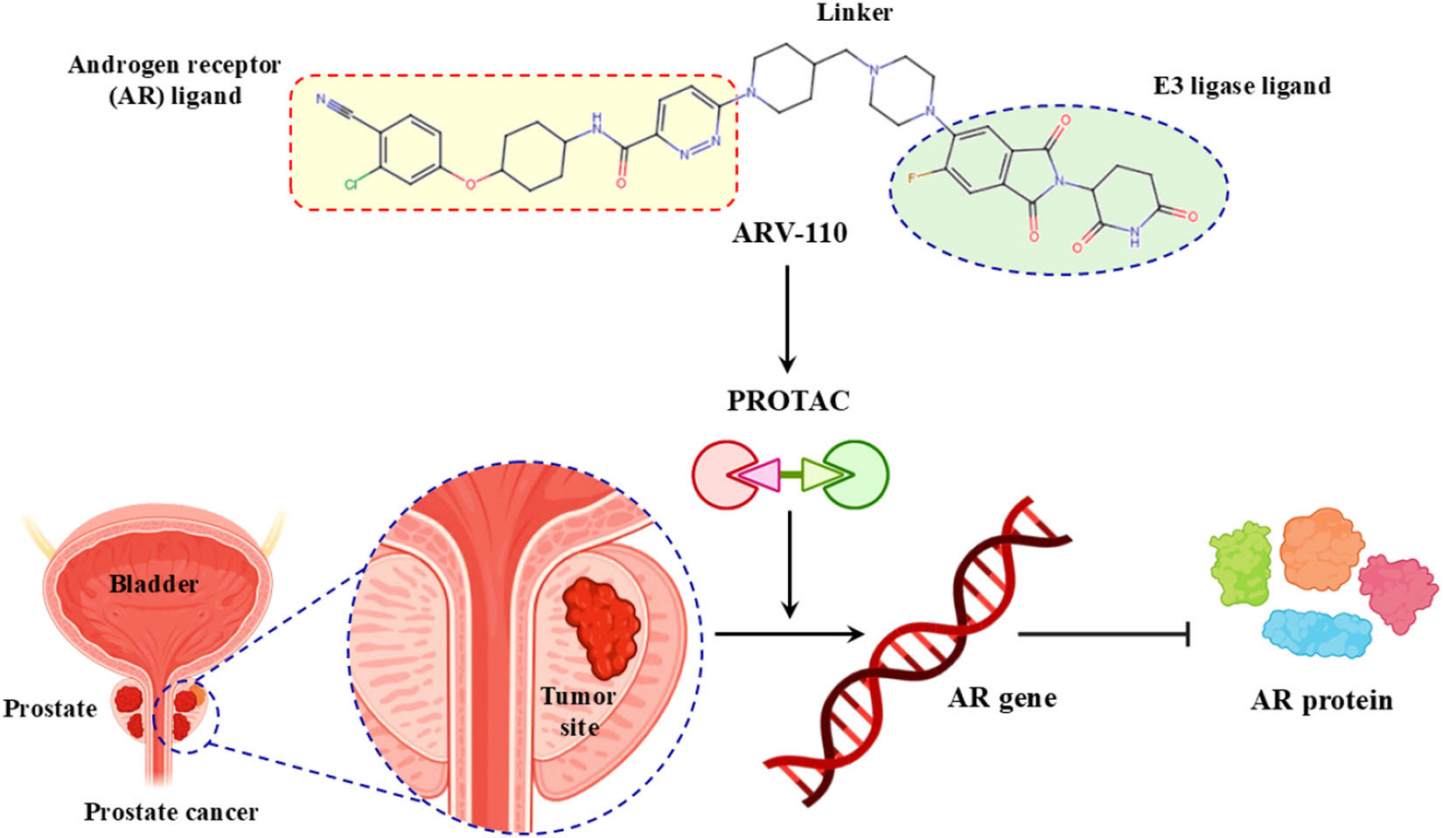
Bavdegalutamide (ARV‐110), a PROTAC targeting androgen receptor (AR) ligand in prostate cancer.

Traditional PROTACs have changed the landscape of drug development by enabling event‐driven degradation of intracellular proteins (Békés et al. 2022). They function through a bifunctional molecule that is comprised of an E3 ligase recruiter, a linker, and a protein of interest (POI) ligand (Liu, Hu et al. 2022). PROTACs induce the ubiquitination and the consequent proteasomal degradation of the target protein through the induction of a ternary complex (Zhao 2024; El‐Hamaky et al. 2025). PROTACs act substoichiometrically; that is, several target molecules are degraded per PROTAC, in comparison with classical inhibitors, which rely on sustained exposure (He et al. 2022). This mechanism has propelled numerous PROTACs into clinical trials, including ARV‐110 for prostate cancer, as clarified in (Figure 7), and ARV‐471 for breast cancer, as a potent and selective means of eliminating disease‐causing proteins (Dubey et al. 2024).

New generation platforms, such as dual‐target PROTACs, TF‐PROTACs, and phosphoTACs, which target previously inaccessible sites and enhance functional selectivity, are rapidly expanding the therapeutic space, in addition to the revolutionary impact already made by conventional PROTACs (Mahajan et al. 2025). These advanced designs offer novel approaches to achieve degradation in a more controlled manner and to manipulate the biological pathway with greater accuracy. Dual‐target PROTACs can achieve synergistic inhibition of oncogenic signals or overcome resistance by degrading two proteins in the same or related pathways simultaneously (Xu, Gu et al. 2024). This cotargeting approach can be achieved by either homo‐PROTACs that degrade dimeric or oligomeric targets or by a single bifunctional molecule that connects two proteins and an E3 ligase. Possible effects include the suppression of compensatory feedback mechanisms and enhanced therapy efficacy in multifactorial diseases, such as cancer (He et al. 2022).

In contrast, TF‐PROTACs go after TFs, one of the most challenging protein classes (Li, Song et al. 2022). They facilitate the degradation of nonenzymatic proteins once regarded as nondegradable by the recognition of a DNA‐binding domain (oligonucleotide‐based approaches) or by interaction with a protein‐protein interaction (ligand‐based approaches) (Zhang et al. 2025). Recent research on TF‐PROTACs has accelerated their development through the application of emerging technologies, including peptide chemistry, DNA‐encoded libraries, and CRISPR‐guided localization. However, the discovery of ligands, as well as intracellular access and delivery, remains the primary challenge to advance their performance further (Wang et al. 2023).

PhosphoTACs enable an additional level of precision by allowing conditional protein degradation based on the phosphorylation status of the proteins (Xiao et al. 2022). Through the regulation of off‐target effects and homeostatic functions, this state‐dependent approach ensures that only a disease‐triggered type of protein will be targeted (Nguyen et al. 2024). Recent progress in developing metabolically stable mimetics, as well as more recent advances toward phosphorylation‐specific degrons, provides new opportunities to target signaling‐dependent diseases (Ou et al. 2025). These three classes of PROTAC further illustrate how protein degradation will develop in the future: precise, context‐sensitive, and flexible for complex biological systems (Madan et al. 2022). From oncogenic TFs to only temporarily active kinases, their combined pathways represent a flexible toolset to tackle many therapeutic challenges (Vicente and Salvador 2024).

## 7 PhosTAC

PhosTACs, phosphorylation‐targeting chimeras, represent a novel category of PROTACs that leverages the codependency of phospho‐dependent bindings for the selective degradation of proteins. PhosTACs use a phosphate‐binding motif or phosphopeptide instead of a traditional E3 ligase ligand, in contrast to classical PROTACs that link a target protein to an E3 ubiquitin ligase using a bifunctional ligand (Zhao and Dekker 2022). This strategy leverages the transient and context‐dependent nature of phosphorylation to trigger protein degradation only when the protein of interest is phosphorylated at specific residues, thereby providing sensitivity that has not been observed previously in the control of protein signaling states (Hu et al. 2023).

PhosTACs leverage existing phosphorylation‐dependent protein–protein interactions (PPIs), such as those established by BRD4‐binding domains, to discriminate between phosphorylation states (Chen, Hu et al. 2021). For example, phosphorylation of BRD4 at sites such as Ser2 or Ser5 generates docking motifs that are presented explicitly to engineered PhosTACs. These modes of action ensure that degradation reactions are active only on the phosphorylated protein, leading to reduced off‐target effects and beneficial therapeutic specificity (Hollingsworth et al. 2023; Gourisankar et al. 2023).

This approach has striking implications in cancer treatment, as aberrant phosphorylation cascades frequently mediate oncogenic signaling (Zhang, Yu et al. 2024). Given that cancer‐specific kinases drive the phosphorylation cascade, killing cancer cells using cancer‐specific phosphorylation‐based degradation of oncogenic proteins is feasible. For example, androgen receptor (AR) variants stabilized by phosphorylation‐dependent interactions are degraded by PhosTAC in prostate cancer models (Kubryń et al. 2025). Additionally, this technique has the potential to overcome drug resistance caused by mutations that preserve phosphorylation states but abrogate ligand binding (Xiao et al. 2022).

**Figure 8:**
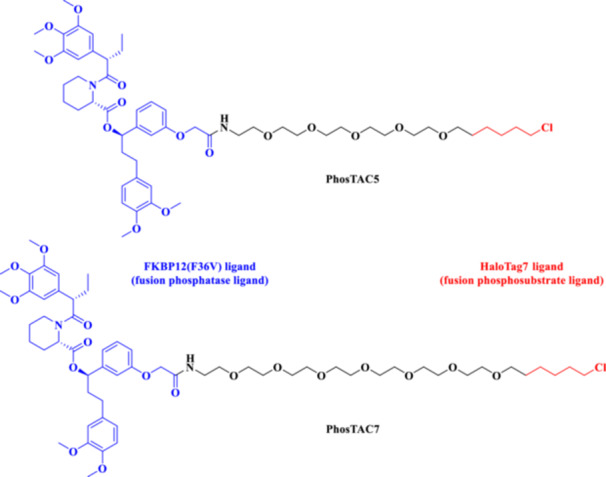
The chemical structures of **PhosTAC5** and **PhosTAC7** as PhosTACs.

**Figure 9:**
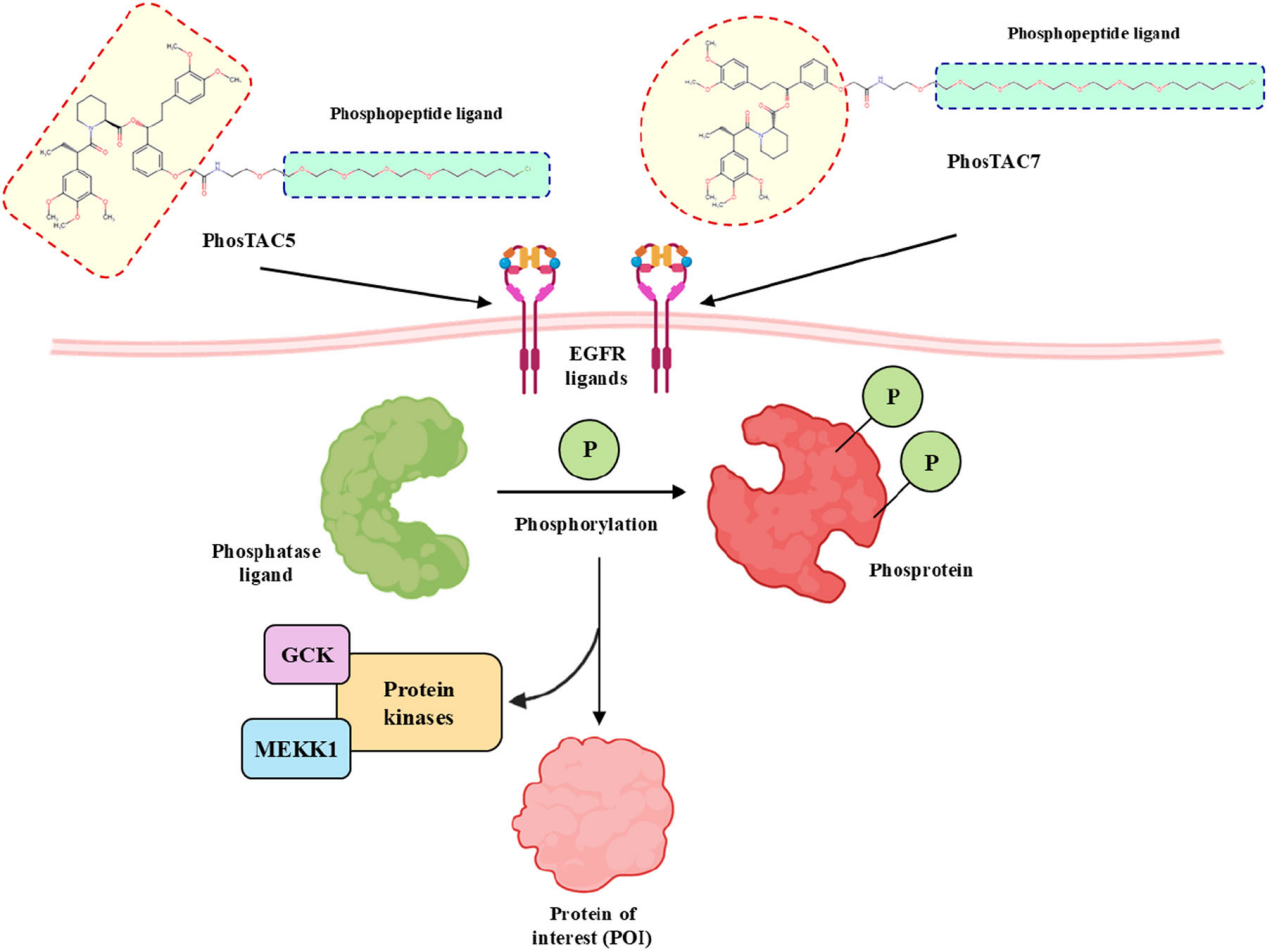
PhosTACs targeting EGFR in cancer cells.

Mechanistically, PhosTACs deviate by replacing the E3 ligase ligand with a phospho‐binding domain, allowing for the linkage of endogenous phosphorylation events to move the target protein into proximity to an E3 ligase, either indirectly or via bound interactions with scaffolds (Hu et al. 2024). This form of molecular logic provides selective degradation and couples cellular signaling state to the degradation decision, allowing for dynamic, context‐dependent therapeutics. As a result, PhosTACs are at the cutting edge of cell‐permeable PROTACs, a new class of target protein degradation technologies (Zhao and Dekker 2022). To demonstrate the viability of rewiring kinase‐substrate relationships in a targeted and selective manner, PhosTAC‐5 and PhosTAC‐7 were developed to recruit the kinases GCK and MEKK1, respectively, to phosphorylate the modified FKBP12 (F36V), as outlined in (Figure 8). PhosTACs specifically promote phosphorylation‐dependent EGFR degradation in cancer cells, providing a new approach to combat resistance mechanisms and to achieve precise spatiotemporal control of oncogenic signaling pathways, as clarified in (Figure 9) (Hu et al. 2024).

## 8 Opportunities and Representative Activity of Kinase Degradation by PROTACs

Kinase‐intended drugs were one of the most successful yet highly mutated drug targets in cancer. However, several traditional kinase inhibitors are plagued by resistance mutations, off‐target toxicity, or limited durability (Li, Gong et al. 2024). PROTACs have been proven to be an effective approach for addressing this challenge by degrading the full‐length kinase protein rather than only inhibiting its catalytic function (Fan et al. 2025). A variety of highly potent PROTACs for targeting various oncogenic kinases have been reported recently. For instance, an EGFR‐targeting PROTAC based on erlotinib and pomalidomide displayed significantly potent antiproliferative activity in A549 cells (IC_50_ = 2.69 ± 0.09 μM) and an overpronounced degrading effect of 96% against EGFR at 72 h time (DC_50_ = 32.9 nM) for induction of prominent G2‐M arrest resulting in apoptosis with activation of caspase‐3 (O. Aboelez et al. 2022). Similarly, the CDK1 PROTAC, appended with a phenyl‐PEG linkage, was also found to exhibit a nanomolar antiproliferative effect in MCF‐7 cells (IC_50_ = 0.10 μM) and enhanced ternary complex formation (ΔG bind = −49.06 Kcal/Mol) compared to this parental compound (Manda et al. 2025). A BTK‐targeting spirooxindole PROTAC was able to induce > 85% degradation of BTK in 6 h in RAMOS lymphoma cells and had efficacy against the C481S mutant form of BTK, a key resistance variant to ibrutinib (IC_50_ = 0.13 μM in RAMOS cells) (Rampeesa et al. 2024). In HER2‐positive models, a phosphorylation‐dependent PI3Kα‐targeting phosphoTAC (i.e., ZM‐PI05) mediated context‐specific depletion of activated kinase pools that resulted in tumor growth inhibition superior to catalytic inhibitors like alpelisib with low on‐target effects in normal tissues (Zhang, Zhang et al. 2024).

## 9 Photocaged PROTAC

Photocaged PROTACs are a groundbreaking leap forward in spatiotemporal manipulation of protein degradation. They are light‐activatable degraders, allowing for fine‐tuned control of protein function by incorporating a photocleavable (or caged) group into the PROTAC structure (Verma and Manna 2020). When exposed to a particular wavelength of light, this photocaging group is cleaved, thereby eliminating the PROTAC, and the E3 ligase that is recruited to the target protein becomes active. This approach enables researchers and clinicians to control degradation with precise temporal and spatial characteristics (in both timing and location). It has negligible off‐target effects, thus potentially enhancing its therapeutic specificity (Xue et al. 2019).

Opto‐PROTACs and pc‐PROTACs are two categories of photocaged PROTACs (Ouyang et al. 2023). In most opto‐PROTACs, genetically encoded or small‐molecule photoswitches, such as azobenzene moieties, which reversibly isomerize between cis‐ and trans‐states upon exposure to light, have been utilized (Cecchini et al. 2021). This conformational change can switch PROTACs on and off. On the other hand, pc‐PROTACs (photocaged PROTACs) depend on the irreversible photolysis of the caging group, most frequently applied as *o*‐nitrobenzyl derivatives, which sterically prevent access to one of the PROTAC's key ligands until photolytic cleavage activates the molecule (Negi et al. 2022). Decaging by light (~365 nm UV‐A) enables the PROTAC to form a normal E3 ligase and a bridge between the E3 ligase and the protein of interest (E3‐POI), re‐engaging the E3 ligase to target the protein for degradation by ubiquitin and the proteasome (Zhu et al. 2021).

This mechanism has since been experimentally proven for BRD4 and the ER, constituting the first light‐induced confined protein degradation in living cells and even in vivo. For example, a photocaged PROTAC against BRD4 was designed in an inactive form and activated with light and a caged ligand, thereby causing the rapid degradation of BRD4, one of the most important epigenetic regulators involved in cancer development (He et al. 2023). The specificity of this system further enables searches of transient protein function within the cell signaling pathway by achieving the pulse‐like light treatment, leading to the degradation kinetics necessary for more detailed experiments (Cheng et al. 2025).

**Figure 10:**
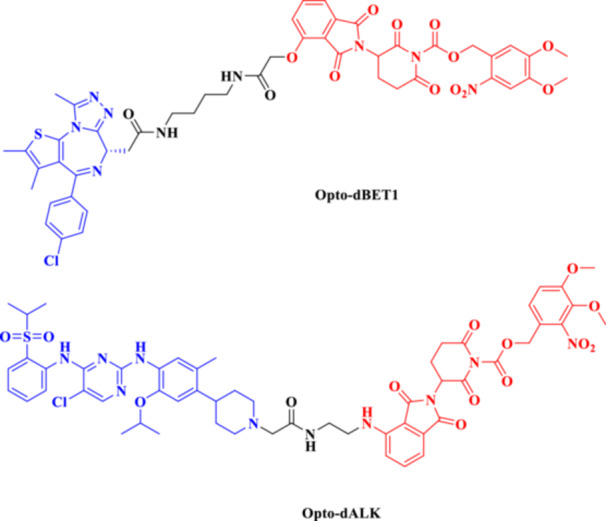
The chemical structures of **Opto‐dBET1 and Opto‐dALK PROTAC** as Photocaged PROTAC.

Photocaged PROTACs, in the context of cancer therapeutics, have the distinct advantage of confining the extent of degradation to cancer cells exposed to light, thus reducing the potential side effects on healthy organs (Xu, Ohoka et al. 2024). This provides opportunities for noninvasive, ligand‐directed, photo‐activatable therapeutics to be used in treating solid tumors or superficial malignancies with high spatial specificity. In addition, the reversibility of opto‐PROTACs allows dynamic control over dose and timing, which is desirable when handling tightly regulated signaling molecules (Reynders and Trauner 2021). Although still in the early stages of development, photocaged PROTACs represent one of the emerging frontiers of conditional degraders, where bioresponsive triggers are combined with synthetic chemistry. Given further advances in light delivery approaches (e.g., near‐infrared imaging or fiber optics), these systems are emerging as promising options for research and therapeutic applications (He et al. 2023; Bhole et al. 2025). Compounds like Opto‐dBET1 and Opto‐dALK have beautifully proved this capability (Figure 10). A 4,5‐dimethoxy‐2‐nitrobenzyl (DMNB) photocage renders the well‐known BRD4 degrader dBET1 inert, resulting in the light‐activatable variant known as opto‐dBET1. The cage is removed when exposed to 365 nm UV light, causing BRD4 to degrade rapidly in living cells with excellent spatiotemporal control. Similar to this, Opto‐dALK targets the oncogenic ALK protein with a photocaged ligand, allowing for selective destruction only when activated by light. These proof‐of‐concept molecules demonstrate that it is possible to accurately control the kinetics of protein breakdown in a light‐dependent manner, which has broad implications for research on signal transduction and targeted cancer therapy.

## 10 Folate‐Caged PROTAC

Folate‐caged PROTACs are a new subgroup of targeted protein degraders, consisting of folic acid (FA‐) moieties and designed to take advantage of the overexpression of folate receptors (FRs), particularly folate receptor alpha (FRα), on the plasma membranes of numerous tumor cells (Liu, Chen, Liu et al. 2021). This approach enables the targeted delivery of the PROTAC molecule to cancer tissues, thereby increasing the therapeutic index and reducing systemic toxicity. The folate portion is a targeting ligand that binds with high affinity to FRα, which is overexpressed in many cancers, including ovarian, breast, and nonsmall cell lung cancers, but is virtually absent in most normal tissues (Ma et al. 2024).

Mechanistically, folate‐caged PROTACs are typically designed as folic acid conjugates at one terminal of the PROTAC scaffold, either directly or through a linker that facilitates correct orientation and binding to the receptor (Lin et al. 2025). After recognition of FRα on the surface of the tumor cell, the whole conjugate is taken up by receptor‐mediated endocytosis (Cheng et al. 2025). After the PROTAC is internalized, it becomes bioaccessible in the intracellular space, where it can interact with the protein target of interest, as well as the recruited E3 ligase, to mediate site‐specific ubiquitination and subsequent proteasomal degradation (Sosič et al. 2022).

**Figure 11:**
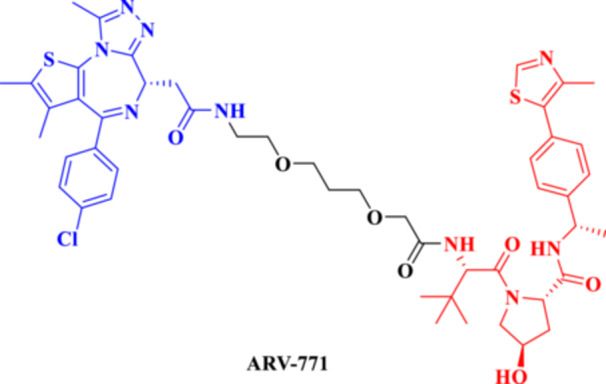
The chemical structure of **ARV‐771** as folate‐caged PROTAC.

**Figure 12:**
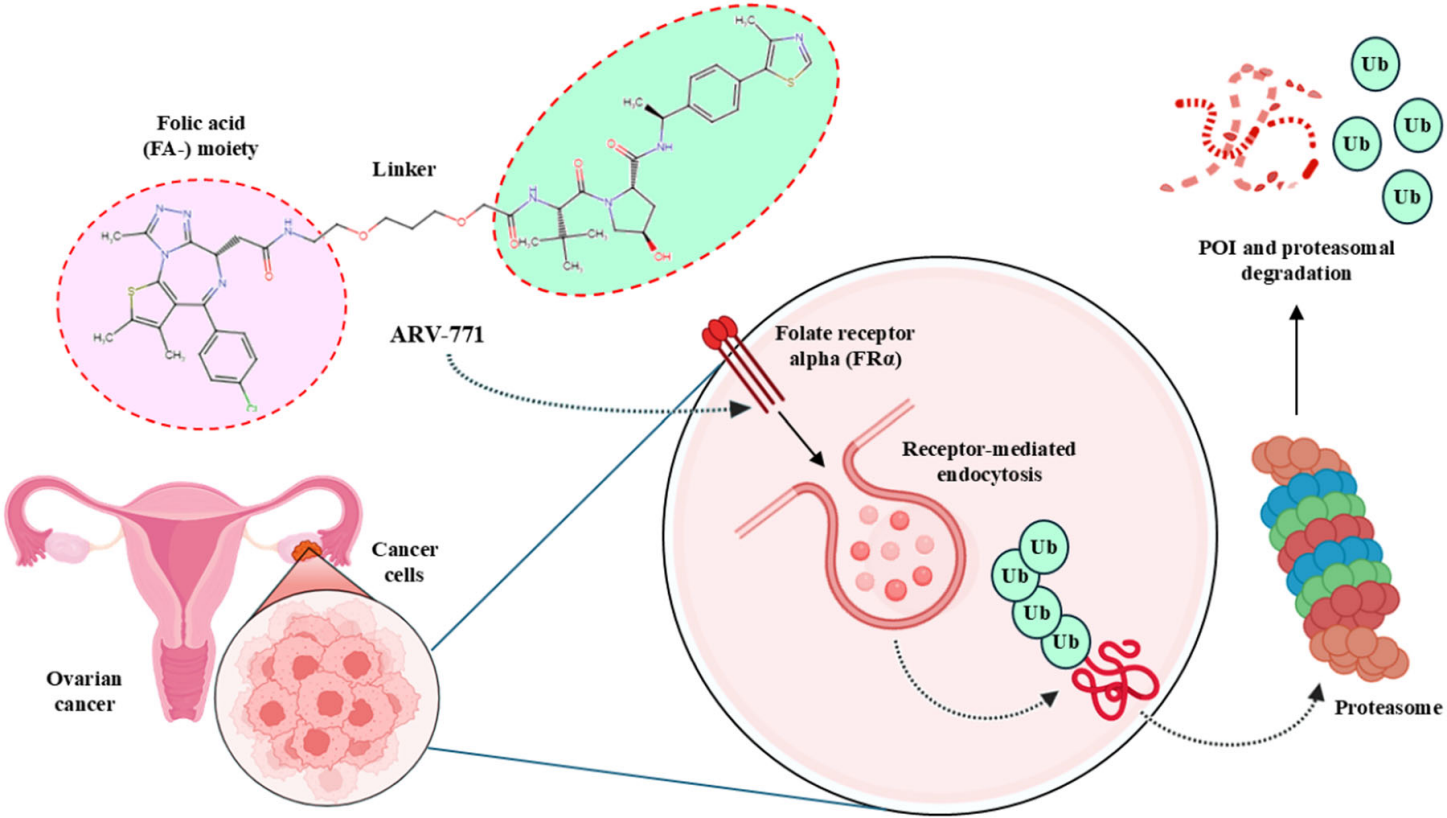
Mechanism of ARV‐771 as folate‐caged PROTAC targeting folate receptor alpha (FRα) ligand in ovarian cancer.

The strategy offers a two‐fold advantage, thanks to its capability for receptor‐mediated targeting and the powerful downstream degradation of PROTACs. One example is the folate‐fused BRD4 degraders, which were reported to be enriched in FRα‐positive tumor models, exhibiting robust on‐target degradation while sparing off‐tumor damage (Chen, Liu et al. 2021). These folate‐targeted PROTACs are of particular interest for the selective ligand‐based degradation of undruggable or transcriptional regulators in cancer cells, while sparing normal tissues—a critical issue in oncology drug design (Zhao and Dekker 2022). In addition, folate‐caged PROTACs are also in good agreement with the emerging trends in tumor‐targeted systems, particularly for solid tumors with receptor expression (Bhole et al. 2025). They are also compatible with theranostic strategies to integrate targeted degradation with imaging modalities via folate conjugation. Although still in a preclinical stage, the folate‐caging approach holds great promise for precision oncology by offering an additional layer of selectivity for PROTAC pharmacology (Yan et al. 2022). Folate‐conjugated ARV‐771 (Figure 11), a well‐characterized BRD4‐targeting PROTAC, is a noteworthy illustration of this tactic. Researchers were able to selectively transport a folic acid moiety to tumor cells that overexpress FRα by attaching it to ARV‐771, as explained in (Figure 12). This allowed for effective BRD4 breakdown in these cells with minimal impact on healthy tissues. With improved tumor selectivity and less systemic exposure, the altered molecule maintained its strong breakdown potential. This design is provided as a proof‐of‐concept for ligand‐directed delivery systems in the context of transcriptional regulator degradation, illustrating how folate‐based targeting may enhance the therapeutic window of PROTACs. Table 1 summarizes some differences between several PROTACs that were discussed in this review.

**Table 1:** PROTACs comparison.

Feature/aspect	Conventional PROTACs	Dual‐target PROTACs	TF‐PROTACs	PhosphoTACs	References
Target type	Single target (typically enzymes, kinases, receptors)	Two proteins (e.g., kinases, epigenetic modulators)	Transcription factors (e.g., MYC, STAT3)	Phosphorylated proteins (e.g., PI3Kα, FRS2α)	Liu, Chen, Kaniskan et al. (2021); Kubryń et al. (2025); Yan et al. (2022)
Targeting strategy	Ligand for POI + E3 ligase ligand	Two ligands for two POIs, linked to one E3 recruiter	Ligand‐based (via cofactors) or oligo‐based (via DBDs)	Phospho‐binders (peptides or mimetics) recognizing the phosphorylated form	Li, Song et al. (2022); Xiao et al. (2022); Zou et al. (2024)
Mechanism of action	Ternary complex formation and ubiquitin‐mediated degradation	Simultaneous ternary complex formation and degradation of both targets	Recruitment of TF or its binding partner to E3 ligase	Phosphorylation‐dependent binding enables conditional degradation	Kubryń et al. (2025); Riching et al. (2022); Li and Crews (2022)
Key advantages	Broad applicability, catalytic mechanism, potent, and selective	Synergistic targeting; resistance suppression	Access to “undruggable” TFs; high sequence specificity	Temporal and signal specificity; kinase‐agnostic degradation	Mahajan et al. (2025); Li, Song et al. (2022); Xiao et al. (2022)
Synthetic complexity	Moderate (2 ligands + linker)	High (2 warheads + linker + E3 ligand)	Moderate to high (especially with oligonucleotide conjugation)	Moderate to high (requires phosphomimetic stability)	Zhang et al. (2025); Meng et al. (2021); Ruffilli et al. (2022)
Representative examples	ARV‐110 (androgen receptor), dBET1 (BRD4)	BRD4/HDAC6 dual degraders, EGFR/HER2	JQ1‐MYC, GAS promoter‐STAT3 PROTAC, ERα‐TF‐PROTAC	pFRS2α degrader, PI3Kα‐selective PhosphoTAC	Kubryń et al. (2025); Zheng et al. (2021); Li, Song et al. (2022)
Challenges	Off‐target effects, resistance, bioavailability	Increased molecular weight, off‐target effects	Delivery, nuclear access, immunogenicity (oligos)	Phospho‐state dependency, degradation of degron itself	Burke et al. (2022); Lin et al. (2025); Martín‐Acosta and Xiao (2021)
Applications	Cancer, immunology, neurodegeneration	Oncology, drug resistance, multinode suppression	Cancer, transcriptional diseases	Cancer, RTK/kinase‐driven diseases, signaling modulation	Békés et al. (2022); Liu, Hu et al. (2022); Kubryń et al. (2025); Zhang et al. (2025); Li, Song et al. (2022); Li and Crews (2022)

## 11 PROTACs Kinases Inhibition

O. Aboelez et al. reported the development of a novel class of **pomalidomide‐based PROTACs** through modularization into an EGFR inhibitor, polyethylene glycol (PEG)‐type linker, and CRBN‐recruiting ligand (**Entry 1,** Table 2). A Finkelstein reaction was used to obtain the key intermediate *N*‐(2‐(2,6‐dioxopiperidin‐3‐yl)‐1,3‐dioxoisoindolin‐4‐yl)‐2‐(2‐iodoethoxy)ethoxy)acetamide and also as the electrophilic anchor for the coupling reactions with quinoxaline and hydrazide derivatives. The final PROTACs were then produced by nucleophilic substitution in moderate to good yields (50%–60%) and purified by flash chromatography. NMR, IR, and other spectral analyses indicated that the real degraders were consistently formulated with an intact architecture, allowing for the fine‐tuning of linker polarity and target affinity (O. Aboelez et al. 2022).

**Table 2:** PROTACs kinase(s) inhibition.

Entry	Structure		Kinase inhibition activity			Anticancer activity				References
**1**	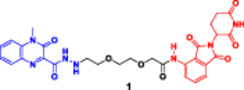		**Enzymes**	**IC**_**50**_ **[µM]**			**IC**_**50**_ **[µM]**			O. Aboelez et al. (2022)
			**(1)**	**Erlotinib**	**Cell lines**	**(1)**	**Erlotinib**	**Doxorubicin**		
			EGFR^WT^	0.10 ± 0.03 µM	0.32 ± 0.05 μM	**HepG2**	3.02 ± 0.12	13.11 ± 1.28	6.22 ± 0.45	
						**HCT116**	3.32 ± 0.15	16.76 ± 1.65	5.52 ± 0.25	
						**MCF‐7**	3.92 ± 0.19	21.76 ± 1.85	7.89 ± 0.55	
			EGFR^T790M^	4.02 ± 0.19 µM	21.44 ± 0.75 µM	**A549**	2.69 ± 0.09	19.33 ± 1.85	NT	
**2**	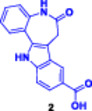 , 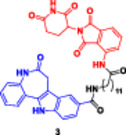		**Enzymes**	**IC**_**50**_ **[µM]**			**IC**_**50**_ **[µM]**			Manda et al. (2025)
				**(4)**	**(3)**	**Cell lines**	**(4)**	**(2)**	**Doxorubicin**	
	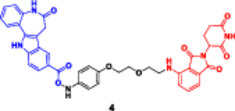		CDK1 in MCF‐7	5.5–16	Marginal decrease	**A549**	0.12	4	3.64	
					**MCF‐7**	0.10	*	0.73		
**3**	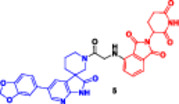 , 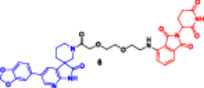 , 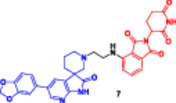	Enzymes	**IC**_**50**_ **[µM]**				**IC**_**50**_ **[µM]**			Rampeesa et al. (2024)
			**(5)**	**(6)**	**(7)**	**Cell lines**	**(5)**	**(6)**	**(7)**	
		BTK (RAMOS)	Null	Null	Decreased at 1x and 2x IC_50_	**RAMOS**	0.13 ± 0.03	0.54 ± 0.14	9.79 ± 3.39	
						**CCRF‐CEM**	0.25 ± 0.03	0.38 ± 0.08	3.81 ± 0.75	
		ITK (JURKAT)	Null	* (not tested)	Null	**JURKAT**	0.73 ± 0.13	48.00 ± 3.41	3.42 ± 0.57	
**4**	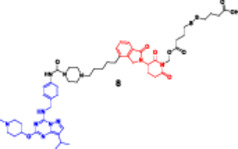 , 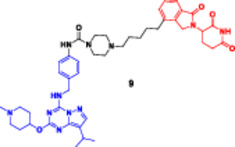	**Enzymes**	**IC**_**50**_ **[µM]**				**IC**_**50**_ **[µM]**			Zou et al. (2024)
			**(8)**	**(9)**		**Cell lines**	**(9)‐NPs**	**Free (9)**		
		CDK9 (MDA‐MB‐231 cells)	0.14 μM	0.04 µM		MDA‐MB‐231 tumor‐bearing mice	CDK9 Reduction ~70%	Lower than 9‐NPs		
			**DC**_**50**_ **[µM]**							
			**Free (9)**	**(9)‐NPs**	Ligand‐free PROTAC prodrug NPs					
			0.081 µM	0.079 µM	0.4029 µM					
**5**	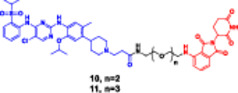	**Enzymes**	**IC**_**50**_ **[µM]**				**IC**_**50**_ **[µM]**			Yan et al. (2021)
			**(10)**	**(11)**	**LDK378**	**Cell lines**	**(10)**	**(11)**	**LDK378**	
		ALK				**H3122**	0.7 ± 0.05	0.3 ± 0.02	1.1 ± 0.21	
			2.3 ± 0.22	1.6 ± 0.12	0.81± 0.07	**H2228**	3.5 ± 0.21	0.9 ± 0.03	1.3 ± 0.14	
						**H1299**	7.59± 0.34	2.84 ± 0.23	2.92 ± 0.19	
						**A549**	2.6 ± 0.11	1.6 ± 0.09	1.32 ± 0.06	
						**HeLa**	2.23 ± 0.09	1.18 ± 0.06	0.94 ± 0.04	
**6**	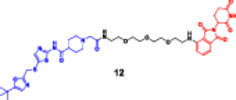	**Enzymes**	**Western blot analysis**			**Cell lines**	**EC**_**50**_ **[nM]**			Noblejas‐López et al. (2022)
							**(12)**			
		CDK9	**(12)**			**Luminal A and B**	Below 100 nM			
			caused a decline in CDK9 in BT474, BT474‐RH, and BT474‐TDM1R cells			**HER2+ and TN**	Higher concentrations to reach EC_50_			
						**BT474 and resistant cells**	50–75 nM			
**7**	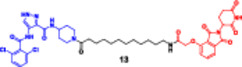	**Enzymes**	**IC**_**50**_ **[nM]**			**Cell lines**	**Cell lysate assay**			Mize (2023)
			**(13)**				**(13)**			
		CDK9	Forty nanomolar represents the concentration required to inhibit 50% of cell viability. Indicates high potency in inhibiting cell growth.			**Molm13**	50–100 nM Measured using antibody stainingfor CDK9			
**8**	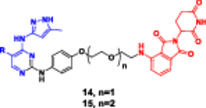	**Enzymes**	**Immunoblotting**			**Cell lines**	**IC**_**50**_ **[µM]**			Manda et al. (2020)
			2‐Chloro‐N‐(5‐methyl‐1H‐pyrazol‐3‐yl)pyrimidin‐4‐amine **(negative control)**	**(14)**|**(15)**			**(15)**	**(14)**		
		IGF‐1R and Src in MCF7 and A549	Not degrading both cells at concentration 5 μM	Src and IGF‐1R degrade at Concentration 1 and 5 μM		**A549**	7.6 μM	4.2 μM		
						**MCF‐7**	2.7 μM	3.3 μM		
**9**	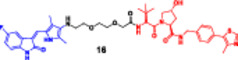 , 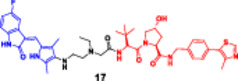 , 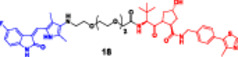	**Enzymes**	**IC**_**50**_ **[µM]**		**Cell lines**	**IC**_**50**_ **[µM]**				Zhai et al. (2022)
			**(16)**			**(16)**	**(17)**	**(18)**	**Sunitinib**	
		FLT‐3/KIT in K562/HL‐60 cells	**K562**	**HL‐60**	**HL‐60**	2.9 ± 1.5b	36.5 ± 3.6	12.7 ± 2.8	8.7 ± 1.0	
			Twenty micromolar of 16 or 17 level of FLT‐3 was reduced	Ten micromolar of 16 reduced the protein level of FLT‐3	**K562**	11.6 ± 4.4	8.4 ± 1.6	15.2 ± 2.6	5.8 ± 1.3	
				Sixteen was higher than 10 μM levels of c‐KIT were significantly reduced	**A498**	13.1 ± 4.5	26.5 ± 5.3	18.1 ± 3.4	1.2 ± 0.3	
**10**	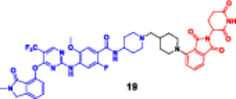	**Enzymes**	**Western blotting**|**DC**_**50**_		**Cell lines**	**IC**_**50**_ **[µM]**				Xu, Gu et al. (2024)
		Total FAK In MDA‐MB‐231 cells	Nineteen degradation of total FAK (27.72 nM) and phosphorylated FAK (60.10 nM)		**4T1**	0.73 μM		3.62 μM		
					**MDA‐MB‐231**	1.09 μM		6.15 μM		
					**MDA‐MB‐468**	5.84 μM		21.07 μM		
**11**	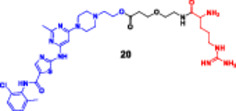	**Enzymes**	**DC**_**50**_ **[nM]**		**Cell lines**	**IC**_**50**_ **[µM]**				Zhang, Ma et al. (2023)
			Arg‐PEG1‐Dasa			Arg‐PEG1‐Dasa				
		BCR–ABL in K562 cells	0.85 nM degrades 50% protein attained 98.8% reduction at 5 nM		**CML K562 cells**	0.3595				
					**K562 CML xenograft mouse**	Tumor size and BCR–ABL levels were significantly downregulated				
**12**	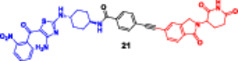	**Enzymes**	**Western blotting**		**Cell lines**	**IC**_**50**_ **[nM]**				Chen et al. (2024)
			**(21)**			**(21)**				
		CDK9|CDK7	CDK9 decreased protein levels, strongest binding affinity	CDK7 strongest binding affinity		72 h		48 h		
					**MCF7**	8.86 nM		28.86 nM		
					**T47D**	21.78 nM		41.67 nM		
**13**	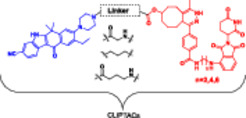	**Enzymes**	**DC**_**50**_ **[nM]**		**Cell lines**	**IC**_**50**_ **[nM]**				Xie et al. (2025)
			**NANO‐CLIPTACs**			**NANO‐CLIPTACs**				
		ALK in H3122 cells	W4@cRGD‐LPs and Z2@cRGD‐LPs ratio of 1:1	175.37 ± 53.24 nM	**H3122**	20.98 ± 1.44 nM				
					**HUVEC/A549**	Nano‐CLIPTACs minimal effects on ALK negative cells				
					**H3122 xenograft mouse**	5 mg/kg W4@cRGD LPs and 4.34 mg/kg Z2@cRGD‐LPs combination was 30 fold higher				
					**Karpas 299**	10 mg/kg W4@cRGD‐LPs + 8.68 mg/kg Z2@cRGD‐LPs, inhibition rate of 77.4%				
**14**	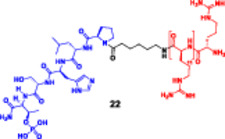	**Enzymes**	**Immunoblotting**		**Cell lines**	**IC**_**50**_ **[µM]**|**MTT assay**				Gunasekaran et al. (2024)
			**(22)**			**(22)**				
		PLK1	Maximum degradation (22) at 5 μM, Dmax of ∼80%	DC_50_ 2.5 μM	**HeLa**	17.98 ± 0.31 μM				
					**A549**	20.63 ± 2.43 μM				
		HeLa cells			**PC9**	17.05 ± 1.46 μM				
					**H1975**	24.45 ± 1.31 μM				
					**Mice tumor**	**(22) (10 mg/kg), PBS Injected through the tail vein**				
		A549	**Western blots**		**HeLa**	**(22) (**3.4‐fold decrease)				
					**PC9**	**(22)** (5.2‐fold decrease)				
					**A549**	**(22)** (6.3‐fold decrease)				
			Slightly reduced degradation, Dmax of more than 60% at 30 μM	DC_50_ at ∼27 μM	**Double‐mutated (L858R/T790M) H1975**	**Combination (22) and osimertinib**				
						Inhibiting tumor growth by approximately sevenfold				
		H1975	Dmax of 95% at 30 μM	DC_50_ at ∼12 μM		**Combination (22) and osimertinib**				
		PC9	(22) at 25 μM, Dmax of 100% at 30 μM	DC_50_ at ∼12 μM		Combined (1,1 ratio), MTT Assay show IC_50_ For combined therapy less than (22) or Osimertinib used individually				
**15**	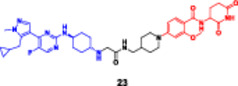	**Enzymes**	**DC**_**50**_ **[µM]**			**Cell lines**	**IC**_**50**_ **[µM]**|**CCK8 assay**			Wang, Jiang et al. (2024)
			**(23)**				**(23)**			
		100 nM treatment in MV4‐11 cells	CK1α	CDK7	CDK9	**MV4‐11**	0.096 ± 0.012 μM			
						**MOLM‐13**	0.072 ± 0.014 μM			
			19.91 nM	19.94 nM	40.94 nM	**MV4‐11**	Western blot	CCK8 assay		
							pretreatment 10.0 μM thalidomide blocked CK1α degradation	IC_50_ 0.096–8.96 μM after pretreatment thalidomide		
**16**	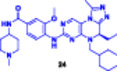 , 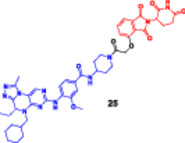	**Enzymes**	**IC**_**50**_ **[nM]**|**[uM]**		**Cell lines**	**IC**_**50**_ **[µM]**				Hu et al. (2022)
			**(24)**	**(25)**		**(24)**		**(25)**		
		PLK1			**MV4‐11**	—		0.003 µM		
					**VCaP**	—		0.016 µM		
			22 nM	0.985 uM	**LNCaP**	—		0.021 mM		
					**22Rv1**	—		0.053 µM		
					**PC‐3**	Better antiproliferative		—		
					**DU145**	than WWL0245				
**17**	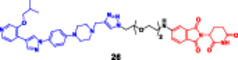	**Enzymes**	**DC**_**50**_ **[uM]**		**Cell lines**	**IC**_**50**_ **[µM]**|**CC**_**50**_ **[µM]**				Sun et al. (2024)
			**(26)**			**(26)**	**JNJ‐47117096**	**MELK‐8a**		
		MELK	2.1 uM with D_max_ 91.6%		**MDA‐MB‐231**	13.8 µM	7.8 µM	2.3 µM		
					**CC**_**50**_ **[µM]**					
					**RAMOS IC**_**50**_ **[µM]**	1.8 µM	9.1 µM	3.4 µM		
**18**	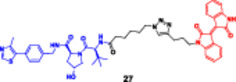	**Enzymes**	**IC**_**50**_ **[µM] | Western blot**		**Cell lines**	**IC**_**50**_ **[µM]**|**MTT assay**				Liu et al. (2024)
			**(27)**			**(27)**				
		ATM	Degradation was dose dependently VHL ligand VH032‐Boc as well as Mei	Cross SW620, SW480, and K562 cells	**A2780**	3.96 ± 0.69				
					**SW13**	3.31 ± 0.25				
					**NCI‐H358**	> 20				
					**SW620**	3.70 ± 0.12				
					**SW480**	2.59 ± 0.12				
					**LOVO**	6.73 ± 0.66				
					**RKO**	3.95 ± 0.11				
					**HCT116**	5.82 ± 0.16				
					**COLO‐205**	5.11 ± 0.06				
					**K562**	4.06 ± 0.19				
		Xenograft Mouse	Decrease of ATM	(27)/AZD6738‐treated mice	**HIEC‐6**	23.93 ± 0.39				
					**SW620 xenograft mouse**	15 mg kg −1 (27) 25 mg kg −1 (AZD6738)		(27) and AZD6738 induced a notable reduction in tumor load		
					**Vivo milieu**	No significant weight loss upon the administration of (27), AZD6738, or their combinatorial regimen				

*Note:* E3 ligase ligand: **Red** color; POI ligand: **Blue** color; Linker: **Black** color.

The bioassay results indicated that compound **(1)** was the most potent anticancer agent against MCF‐7, HepG‐2, HCT‐116, and A549 cell lines with IC_50_ values of 3.92 ± 0.19 μM, 3.02 ± 0.12 μM, 3.32 ± 0.15 μM, and 2.69 ± 0.09 μM, respectively. It exhibited higher potency than erlotinib and doxorubicin and showed good EGFR^WT^ (IC_50_ = 0.10 ± 0.03 μM) and EGFR^T790M^ (IC_50_ = 4.02 ± 0.19 μM) kinase inhibitory activity. Western blotting results demonstrated EGFR degradations (Dmax = 96%) in A549 at 72 h (DC_50_ = 32.9 nM). In addition, compound **(1)** also caused prominent G2‐M cell cycle arrest and apoptosis in all cell lines examined, which increased caspase‐3 protein by 6.31‐fold in HepG‐2 cells, demonstrating strong induction of apoptotic signal (O. Aboelez et al. 2022).

Manda et al. demonstrated that **paullone‐derived PROTACs** can be synthesized by HATU‐mediated amide coupling of 6‐oxo‐5,6,7,12‐tetrahydrobenzo (Kamaraj et al. 2024; Liu, Hu et al. 2022) azepino[4,5‐b]indole‐9‐carboxylic acid **(2)** with E3 ligase ligand (thalidomide and VHL) through linear or aromatic‐PEG linkers (**Entry 2,** Table 2). The key intermediates prepared were azepinone‐functionalized thalidomide amines and substituted PEG azides, which were further converted into PROTACs **2**, **3**, and **4**. The yield and biological activity were highly dependent on structural optimization of the linker length and rigidity. For example, PROTAC **4** carrying a phenyl‐substituted PEG linker afforded improved physicochemical properties and docking affinity (ΔG_bind = −49.06 kcal/mol) with CDK1 superior to the associated paullone parent inhibitor (ΔG_bind = −20.84 kcal/mol) (Manda et al. 2025).

Among the synthesized PROTACs, compound **(4)** was represented as one of the potent CDK1 degraders. In the cytotoxicity assay, compound **(4)** suppressed the proliferation of MCF‐7 breast cancer cells with an IC_50_ of 0.10 µM and lung cancer A549 cells with an IC_50_ of 0.12 µM, which were better than those of the reference drug doxorubicin (IC_50_ = 0.73 µM and 3.64 µM, respectively). Dose‐dependent CDK1 degradation in MCF‐7 cells was observed between 5.5 and 16 µM, as determined by Western blot, validating the proteasome‐mediated engagement of the target. Compounds **(2)** and **(3**) also exhibited moderate antiproliferative activity against the MCF‐7 cells, with IC_50_ values of 5.3 and 6.6 µM, respectively. The phenyl‐PEG linker in compound **(4)** may be responsible for further promoting the formation of the ternary complex and selective protein degradation, particularly highlighting its potential in targeted cancer treatment (Manda et al. 2025).

Rampeesa et al. described a new **azaspirooxindolinone‐based PROTAC**, which originated from a small molecule BTK inhibitor by connecting the small molecule with a CRBN ligand (pomalidomide) with the aid of a short PEG linker (**Entry 3,** Table 2). The spirooxindole framework was prepared in a multi‐step sequence that included cyclization between isatin‐based intermediates and alkylation with piperidine derivatives. The warhead was conjugated first to a PEG chain and, in turn, to pomalidomide through carbodiimide coupling and amide coupling, respectively. The compound was also characterized using NMR and LC‐MS. The design focused on BTK interaction and efficient ternary complex formation, with the linker length specifically adjusted for maximal degradation potency and high binding affinities to both BTK and CRBN (Rampeesa et al. 2024).

The developed spirooxindole‐based PROTACs effectively inhibited and degraded BTK selectively with a biochemical IC_50_ value of 0.25 ± 0.03 µM, and this was translated to potent cellular activity in RAMOS lymphoma cells (IC_50_ = 0.50 µM) and the absence of cytotoxicity against the normal HEK293 cells (IC_50_ > 10 µM), demonstrating selective antitumor activity against B‐cell malignancies. It was also demonstrated that rational linker design is a crucial parameter for efficient PROTAC activity compared to other derivatives, as shown by PROTAC **5** (IC_50_ = 0.13 ± 0.03 µM in RAMOS) and PROTAC **6** (IC_50_ = 0.54 ± 0.14 µM in RAMOS). Western blot analysis demonstrated the degradation of BTK with more than 85% knockdown after 6 h. When compared with conventional BTK inhibitors, this spirooxindole‐based PROTAC retained activity against the C481S‐mutant BTK, a frequently acquired resistance mutation. This dual mechanism of action, involving kinase inhibition and proteasomal degradation, enhances its candidacy as a novel class of next‐generation BTK‐targeting agent in hematological malignancies, including those that are covalently resistant, such as ibrutinib (Rampeesa et al. 2024).

Zou et al. described a synthetic route of tumor‐targeted PROTACs (VHL‐ and CRBN‐based PROTACs) that utilizes ester link formation between a redox‐converting dithiodibutyric acid linker and PEG‐b‐PLGA. They demonstrated that reduction of the linker provided triggered release of PROTACs in the tumor microenvironment (**Entry 4,** Table 2). For example, ARV‐771 was derivatized with a γ‐disulfide linkage to obtain **DT‐ARV‐771**, resulting in a more potent intracellular activator. The nanoprecipitation of CRGDK‐modified PEG‐b‐PLGA and PROTAC‐polymer conjugated to stable spherical nanoparticles (70–80 nm). The nanoparticles (RPG7, RPGD43) exhibit efficient disulfide cleavage in the presence of reducing agents, as verified by HPLC, and are stable in physiological buffers. This modular chemical scaffolding enables the controlled, tumor‐triggered degradation of BRD4 and CDK9, with potential therapeutic implications, including the possible use in combination treatments (Zou et al. 2024).

The designed PROTAC prodrug nanoparticles presented strong antitumor efficacy toward triple‐negative breast cancer (TNBC) models. VHL‐mediated RPG7‐NPs targeting BRD4 demonstrated a DC_50_ of 0.14 μM and an IC_50_ of 0.25 μM, which was more potent than free ARV‐771 (IC_50_ = 0.51 μM) and PG7NPs (IC_50_ = ~0.72 μM). CDK9‐Targeting **(9)‐NPs** Based on CRBN showed a DC_50_ of 0.079 μM in comparison to 0.081 μM for **(9)**, supported by increased CDK9 and c‐Myc degradation and lower IC_50_ at 0.14 μM versus **(9)‐**NPs. Importantly, RPGD43 (3.0 mg/kg) caused significant tumor regression with little toxicity, in contrast to **(9)**, which was lethal at that dose. By combining RPG7‐DOX NPs, the IC_50_ value was decreased to 0.036 μM, and the combination treatment synergistically induced the activity of caspase‐3 as well as the complete eradication of tumors in vivo (Zou et al. 2024).

Yan et al. stated that the **ALK‐targeting PROTAC** was synthesized by connecting alectinib (an FDA‐approved ALK inhibitor) and a pomalidomide‐based CRBN ligand with a medium‐length PEG adaptor to improve flexibility and solubility. The synthesis was carried out using common amide coupling methodology, along with orthogonal protection groups, to ensure the active position of the kinase inhibitor and E3 ligase ligand. The design aimed to maintain affinity to alectinib, as well as form the trimer complex with CRBN (**Entry 5,** Table 2). The PEG linker was chosen based on SAR studies, which had shown enhanced degradation efficiency and cellular permeability. The **ALK‐directed PROTAC** was identified by NMR and HRMS and exhibited high stability in biological environments (Yan et al. 2021).

PROTAC **11** demonstrated potent antitumor activity in various tumor cells, particularly in ALK‐rearranged H3122 cells. It exhibited a potent IC_50_ of 0.3 ± 0.02 µM in H3122, demonstrating a potency superior to that of its warhead, LDK378 (IC_50_ = 1.1 ± 0.21 µM). The IC_50_ values of PROTAC **11** for H2228 and H1299 cells were 0.9 ± 0.03 μM and 2.84 ± 0.23 µM, respectively. Western blotting confirmed that the degradation of ALK fusion proteins was dose‐ and time‐dependent, with nearly complete degradation achieved at 200 nM after 24 h. Moreover, PROTAC **11** had selective cytotoxicity toward H3122 cells with negligible toxicity to normal LO2 hepatocytes and achieved 48% TGI in H3122 xenografts at 50 mg/kg in vivo. These results identify PROTAC **11** as an exciting lead compound for ALK‐specific cancer treatment (Yan et al. 2021).

Noblejas‐López et al. reported that compound **(12)** is a novel generation PROTAC, consisting of the selective CDK9 kinase inhibitor SNS‐032 linked through polyethylene glycol (PEG) to a thalidomide‐based E3 ligase ligand (**Entry 6,** Table 2). This dual activity of the molecule uses the cereblon (CRBN) E3 ubiquitin ligase to mediate the CDK9‐directed proteasomal degradation. As such, the synthetic approach enables the formation of an amide bond linking SNS‐032 to a thalidomide derivative, while maintaining an optimal length and flexibility of the linker for stable ternary complex formation. Structural optimization enhanced pharmacodynamic potency while maintaining selectivity, particularly against CDK7, CDK1, and CDK2, resulting in reduced off‐target kinase inhibition. This structure‐based rational design demonstrates the advantages of ligand‐linker‐E3 in precision oncology‐targeted therapeutics development (Noblejas‐López et al. 2022).

Compound **(12)** effectively inhibited the growth of ER‐positive and ER/HER2 double‐positive breast cancer cells. The in vitro assays showed EC_50s_ of 300 nM in the HER2+/ER− lines (SKBR3, HCC1569, HCC1954). BT474‐derived trastuzumab‐resistant sublines, BT474‐RH and BT474‐TDm1R, were sensitive to SAP30 with EC50s of 50–75 nM, strikingly 10‐fold lower than the HER2‐targeting drug trastuzumab, implying the applicability of SAP30 in overcoming resistance to HER2‐directed therapy. Mechanistically, compound **(12)** promotes CDK9 degradation, increases apoptosis (Annexin V(+), PARP cleavage, caspase‐3 activation), and enhances γH2AX signaling. However, its therapeutic index was poor due to in vivo toxicity, particularly in the gastrointestinal epithelium. Together, these results identify CDK9 degradation as an attractive but delivery‐hurdled strategy for luminal B/HER2+ breast cancers (Noblejas‐López et al. 2022).

Mize described compound **(13)** as a potential PROTAC candidate that had been synthesized in a convergent manner; the thalidomide‐based E3 ligand recruiter was linked to the kinase‐targeting warhead by an optimized PEG linker (**Entry 7,** Table 2). A synthetic route involved nucleophilic aromatic substitution of a bromine‐containing glutarimide derivative to install a primary amine side linker, followed by amide coupling to the kinase inhibitor with EDC/HOBt activation. Preparative HPLC was used to purify the final product, and NMR and HRMS confirmed its structure. The flexible linker length was adjusted for successful ternary complex formation, which guaranteed the correct separation between POI and E3 ligase complex for effective induced proximity, a cornerstone for rational design of PROTACs (Mize 2023).

The EGFR kinase‐targeted PROTAC series inhibited EGFR kinase potently, with a biochemical IC_50_ of 0.42 µM, and displayed a submicromolar antiproliferative IC_50_ value (0.60 µM) in A431 epidermoid carcinoma cells, showing considerable cytotoxic character. The compound **(13)** was active against EGFR L858R/T790M mutants, thus indicating its resistance‐overcoming potential. Western blot analysis indicated more than 80% degradation of EGFR within 8 h. Target dependency was confirmed by the observation of no degradation or cytotoxicity in EGFR‐null control cells. In addition, the repression of the downstream pathways of pAKT and pERK suggested an efficient abrogation of survival signaling. In contrast to the parent EGFR inhibitor, the PROTAC series EGFR kinase degraders had more durable target suppression and increased apoptosis induction, which suggests that this class of agents should have therapeutic relevance in EGFR‐dependent tumors (Mize 2023).

Manda et al. reported the parallel development of **dual degraders of IGF‐1R and Src,** compounds **(14)** and **(15)**, which were synthesized in a convergent manner by linking a common warhead (*N* ^*2*^‐phenyl‐*N* ^*4*^‐(1*H*‐pyrazol‐3‐yl)pyrimidine‐2,4‐diamine) to pomalidomide with a PEG linker (**Entry 8,** Table 2). The synthetic sequence involved azidation of polyethoxyethanols, nucleophilic substitution with 4‐fluoronitrobenzene, azide reduction, amide coupling with thalidomide analogs, and ultimate attachment to kinase inhibitors. Conditions for yields (57% maximum) and linker length formulations enhance recruitment to E3 ligases. The application of CuAAC (click chemistry) also facilitated the synthesis of modular triazole‐based conjugates, highlighting the synthetic flexibility in the design of selective heterobifunctional degraders (Manda et al. 2020).

Compounds **(14)** and **(15)** exhibited a strong anticancer effect by dual degradation of both IGF‐1R and Src in MCF7 and A549. Cell proliferation assays revealed that the antiproliferative activity was dose‐dependent, yielding IC_50_ values of 3.3 and 2.7 µM in MCF7 and 4.2 and 7.6 µM in A549, respectively. Western blot analysis also revealed a marked degradation of IGF‐1R and Src at 5 μM. The wound healing and transwell invasion assays showed a pronounced inhibitory effect on cell migration and invasiveness, a result reinforced by the decreased size and number of colonies in the soft agar assay. These results provide direct evidence that **(14)** and **(15)** are efficient PROTACs for dual kinase degradation, potentially treating drug‐resistant breast or lung cancer (Manda et al. 2020).

Zhai et al. claimed that a library of twelve new PROTACs was developed, linking a VHL ligand to a **Sunitinib** warhead and various linker groups, including PEG chains and heteroatoms. Amidation coupling was carried out by incorporating EDCI/HOBt or HATU, resulting in a stable amide bond between amines and carboxylic acids (**Entry 9,** Table 2). For example, PROTAC **16** was prepared by DMF‐mediated coupling of a PEG linker to the VHL‐ligand and sunitinib at room temperature. Activity was strongly influenced by linker length and composition; PEG linkers resulted in more favorable antiproliferative profiles than those based on an alkyl linker. Through structure optimization, it was found that a two‐unit PEG linker in PROTAC 16 facilitates the efficient formation of a ternary complex, which may contribute to improved ubiquitination efficiency and target engagement (Zhai et al. 2022).

Sunitinib‐based PROTACs potently inhibited the proliferation of leukemia cells by redirecting sunitinib‐induced cytotoxicity, associated with the degradation of FLT‐3 and c‐KIT, two critical kinases implicated in hematologic malignancies. Sunitinib‐based PROTAC scaffold analogs showed very effective anti‐mitotic activity. One scaffold analogue had an IC_50_ of 2.9 ± 1.5 µM, 11.6 ± 4.4 µM, and 13.1 ± 4.5 µM in HL‐60, K562, and A498 cells, respectively. Western blotting results further demonstrated that the levels of FLT‐3 and c‐KIT proteins were significantly decreased in HL‐60 cells after treatment with sunitinib‐based PROTACs. Mechanistic studies revealed that the degradation of FLT‐3 followed a UPS‐dependent pathway, whereas c‐KIT depletion was not UPS‐mediated. On the other hand, PROTAC **17** was more potent against K562 (IC_50_ = 8.4 ± 1.6 μM) and less potent against HL‐60. Selectivity tests showed that both compounds were highly cytotoxic to normal cells (NCM460, IC_50_ > 100 μM). These results demonstrate the potency of PROTAC 9 as a dual‐target kinase degrader for killing leukemia cells and treating resistance (Zhai et al. 2022).

Xu et al. reported that the **FAK‐targeting PROTAC 19** was synthesized by conjugating the clinical FAK inhibitor IN10018 (F1) with a CRBN E3 ligase ligand (thalidomide) via a rigid linker to enhance oral bioavailability (**Entry 10,** Table 2). The molecule (M^+^H^+^ = 928.30 m/z) was confirmed via NMR and LC‐MS. Structural optimization also resulted in an effective ternary complex formation with residues derived from FAK (GLU506, ILE428, MET499) and CRBN (TRP382, PRO354), allowing proteasomal degradation. The design strategy used conformational stability to modulate intracellular stability and degradation kinetics. **(19)** had nanomolar degradation potencies (DC_50_ = 27.72 nM for total FAK, 60.10 nM for p‐FAK), underscoring the importance of structure‐based PROTAC engineering in the design of next‐gen therapeutics (Xu, Gu et al. 2024).

**(19)** demonstrated better anticancer potential than its warhead IN10018 (F1). In 4T1, MDA‐MB‐231, and MDA‐MB‐468 cells, as well as in MDA‐MB‐435, the IC_50_ of **(19)** was 0.73, 1.09, 5.84, and 3.05 μM, respectively, in comparison to values obtained with F1 (3.62, 6.15, 21.07, and 7.20 μM). Moreover, **(19)** could also reverse the multidrug resistance (MDR) of HCT8/T, A549/T, and MCF‐7/ADR cells. More interestingly, **(19)** plus PTX decreased IC_50_ in HCT8/T from 3.22 to 0.020 μM (158.4‐fold reversal) and VCR IC_50_ in A549/T from 1.39 to 0.014 μM (99.3‐fold reversal). Molecularly, **(19)** not only degraded P‐gp but also suppressed ERK/AKT signaling and reduced the levels of EMT markers, such as N‐cadherin, slug, and snail, indicating its dual functions for protein degradation and signal inhibition. These findings suggest **(19)**'s potential as an anticancer and MDR‐reversal drug in the clinic (Xu, Gu et al. 2024).

Zhang et al. highlighted that compound **(20)** is representative of an N‐end rule‐derived PROTAC with a degradative arginine residue, a PEG1 linker, and the BCR–ABL–binding ligand dasatinib. The synthesis starts with an Fmoc‐protected amino acid coupled to a monodisperse PEG linker with a standard peptide coupling agent (e.g., HATU/DIPEA), which is then followed by N‐terminal deprotection (**Entry 11,** Table 2). The dasatinib warhead is linked by an amide bond to the PEG‐amino acid conjugate. Final purification is carried out by RP‐HPLC, and > 95% purity is obtained. Employment of a single PEG unit allowed for tuning of linker length, solubility, and intracellular bioavailability level, which are essential parameters for balancing thedegradation potency and the steric hindrance inside the E3 ligase‐substrate complex (Zhang, Ma et al. 2023).

Compound **(20)** efficiently degrades the BCR‐ABL oncoprotein by the ubiquitination‐proteasome system in K562 chronic myeloid leukemia cells. This agent has an in vitro DC_50_ of 0.85 nM for BCR‐ABL degradation and 98.8% degradation at 5 nM. Its antiproliferative activity is high, with the IC_50_ value of 0.3595 nM (cell counting Kit‐8 assay). Comparative studies of analogs carrying different amino termini (N‐Lys, N‐Leu, N‐Phe) reveal that the Arg‐based PROTAC exhibits better degradation and antiproliferative activities. In vivo, **(20)** markedly suppressed tumor growth in a K562 xenograft mouse model at 10 mg/kg and exhibited no apparent toxicity. These findings highlight the potent, long‐lasting action of this compound, serving as a strong guide for the development of next‐generation anticancer PROTACs targeting oncogenic kinases (Zhang, Ma et al. 2023).

Chen et al. tested that the **PROTAC 21** is a structure‐based designed PROTAC consisting of a selective CDK9 inhibitor warhead (from BAY‐1251152) connected to a pomalidomide‐based CRBN ligand by a triethylene glycol (PEG_3_) linker (**Entry 12,** Table 2). Palladium‐catalyzed coupling was employed for the installation of the heteroaryl core, and amide bond formation was utilized for attachment of the linker to the CDK9 warhead. Reverse‐phase HPLC purified the resultant PROTAC after the final conjugation step. NMR and HRMS confirmed structural identity and composition. The linker was designed to favor productive ternary complex formation between CDK9, PROTAC, and CRBN, thus allowing for both efficient target binding and degradation in ER^+^ breast cancer cells (Chen et al. 2024).

PROTAC **21** exhibited high sensitivity and selectivity in an antitumor assay on ERα‐positive breast cancer cell lines. It displayed an IC_50_ of 28.86 nM in MCF‐7 and 41.67 nM in T47D, which were more potent than L027, L062, and L063, the structurally related analogs. PROTAC **21** also induced G2/M arrest of the cell cycle, markedly inhibited DNA synthesis, and downregulated several oncogenic proteins, including CDK4, CDK6, Cyclin D1, c‐Myc, and Mcl‐1. Mechanistically, it destabilized CDK9 through a CRBN‐dependent ubiquitin‐proteasome pathway. *In vivo*, PROTAC **21** induced the regression of MCF‐7 and T47D xenograft tumors, with no evidence of systemic toxicity. It exhibited more potent effects and higher specificity than those of the available CDK9 inhibitors; thus, PROTAC **21** is a potential candidate for future development of a therapeutic agent for hormone receptor‐positive breast cancer (Chen et al. 2024).

Xie et al. announced that the **Nano‐CLIPTACs** system employs bioorthogonal inverse electron demand Diels–Alder (IEDDA) chemistry to drive in situ self‐assembly of PROTACs that target ALK. Synthetic design began with the conjugation of a trans‐cyclooctene (TCO) arm to an alectinib‐derived ALK ligand (W4) and a tetrazine (TZ) appendage to a pomalidomide‐inspired CRBN ligand (Z2) (**Entry 13,** Table 2). When mixed in tumor microenvironments, W4 and Z2 assembled quickly to form full PROTAC (WZ42) with a conversion rate of > 81.9% within 15 min. Liposomal encapsulation with cRGD‐functionalized DSPE‐PEG2000 resulted in improved receptor‐specific tumor delivery. This modular construct reduces systemic reactivity, avoids off‐target interactions, and enhances pharmacokinetics by fragmenting the high molecular weight structure into drug‐like precursors (Xie et al. 2025).

In H3122 cells, the W4‐Z2 CLIPTAC system displayed strong degradation of the oncogenic fusion protein EML4‐ALK32 (DC_50_ = 359.03 ± 48.29 nM). Compared to the nonencapsulated form, the endocapsulation of these cRGD‐liposomes as Nano‐CLIPTACs had an optimized degradation potential (DC_50_ = 175.37 ± 53.24 nM). The system did not suffer the notorious hook effect observed for traditional PROTACs and maintained the activity at 10 µM. In proliferation studies, W4 and Z2 were used in co‐treatments (1:1), inducing an IC_50_ of 0.68 ± 0.02 µM, which was further potentiated to 20.98 ± 1.44 nM upon encapsulation into liposomes. There was little cytotoxicity in the ALK‐negative cells, indicating that its cell‐killing activity was specific to cancer cells. *In vivo*, Nano‐CLIPTACs inhibited the tumor growth of H3122 and Karpas 299 xenografts without apparent toxicity (Xie et al. 2025).

Gunasekaran et al. demonstrated a new N‐degron‐based strategy in which a **new PROTAC** exploits a new **N‐degron** system based on poly‐arginine (poly‐arginine) to induce the degradation of polo‐like kinase 1 (PLK1), a key driver of mitosis and tumor progression (**Entry 14,** Table 2). The molecule is constructed by connecting a PLK1‐targeting ligand (BI2536 analog) to a poly‐arginine N‐degron motif via a cleavable ester linker. This design exploits the cellular ubiquitin‐proteasome system through the UBR‐box N‐recognin pathway in contrast to classical E3 ligase recruitment, such as that used by PROTACs. The synthesis protocol included a stepwise peptide synthesis, esterification, and HPLC purification to retain functionally intact functional groups and maintain aqueous solubility. Structural validation using MALDI‐TOF‐MS and NMR confirmed the final construct and revealed a novel degron mechanism for kinase‐directed degradation (Gunasekaran et al. 2024).

The new PROTAC exhibited strong PLK1 degradation and potency towards antiproliferative effect in HeLa cells (IC_50_ = 17.98 ± 0.31 μM), A549 cells (IC_50_ = 20.63 ± 2.43 μM), PC9 cells (IC_50_ = 17.05 ± 1.46 μM), H1975 cells (IC_50_ = 24.45 ± 1.31 μM), and its degradation mechanism was confirmed to depend on the N‐degron pathway, exploiting UBR‐box‐harboring ligases instead of utilizing well‐studied classic E3 ligase recruitment. Function assays demonstrated that the treatment prominently induced G2/M arrest of the cell cycle, caspase activation, and apoptosis. Remarkably, this compound selectively destabilized PLK1 relative to PLK2 and PLK3, indicating its target selectivity. In vivo administration of the PROTAC resulted in a marked (75%) reduction in PLK1 levels and tumor volume in a HeLa xenograft, with no apparent systemic toxicity. These results position new PROTACs at the forefront of degradable kinases via a nontraditional degron system, highlighting new opportunities for targeting kinases considered undruggable (Gunasekaran et al. 2024).

Wang et al. described a rationally designed **PROTAC** developed for the dual degradation of CK1α and CDK7, two key kinases involved in transcriptional regulation and cell cycle control. A PEG‐based linker is used to crosslink SR‐3029 (a CK1α/CDK7 dual inhibitor) to a CRBN E3 ligase ligand (**Entry 15,** Table 2). The synthesis session employed conventional amide coupling methods and linker length optimization to enhance the stability of the ternary complex. SAR studies revealed the significance of linker flexibility for the co‐degradation effect. They were analytically characterized by LC‐MS and NMR, demonstrating structural integrity and homogeneity; their in silico docking suggested that both the kinases and the E3 ligase components could be effectively targeted (Wang, Jiang et al. 2024).

PROTAC **23** showed markedly potent antileukemic activity in MV4‐11 and MOLM‐13 AML cell lines (IC_50_ = 0.096 ± 0.012 μM for MV4‐11, and 0.072 ± 0.014 μM for MOLM‐13). Mechanistically, PROTAC **23** effectively decreased the expression of CK1α (DC_50_ = 19.91 nM), CDK7 (DC_50_ = 19.94 nM), and CDK9 (DC_50_ = 40.94 nM), concomitant with an increase in p53. These conditions led to the induction of apoptosis (76% of apoptotic cells vs. 68.6% with A86) and activation of caspases‐3/7. In addition, PROTAC **23** also inhibited the oncogenes MYC, MCL‐1, BCL‐2, and MDM2, as well as the pro‐inflammatory cytokines TNF‐α (IC_50_ = 187.9 nM), IL‐1β (IC_50_ = 80.7 nM), and IL‐6 (IC_50_ = 127.2 nM) in PBMCs, indicating the AML drug's nature (Wang, Jiang et al. 2024).

Hu et al. focused on **ARV‐771**, a selective **BRD4‐targeted PROTAC** that was generated by linking the triazolodiazepine‐based BET inhibitor to a Cereblon (CRBN) ligand through a PEG‐based tether (**Entry 16,** Table 2). The design has the property that the parent inhibitor's affinity with the bromodomain is retained during efficient engagement with CRBN, allowing degradation via the proteasome. Synthesis of **(25)**, the dual BET/PLK1 inhibitor **(24)**, was functionalized via conjugation to a CRBN ligand at the flexible linker conjugation to a CRBN ligand at the piperidine N‐position. The synthesis was based on a multistep solution‐phase chemistry, including click reactions and amide bond formation for the synthesis of the linker. The linker length and polarity were further optimized through SAR studies to enhance ternary complex formation and nuclear permeability. Structural verification by NMR and MS confirmed the molecule's configuration, providing a rationale for its ability to degrade BRD4, as well as its indirect regulation of kinase‐induced oncogenic pathways (Hu et al. 2022).

ARV‐771 affects kinase‐dependent oncogenic signaling indirectly by inducing the strong degradation of BRD4, a master regulator of transcriptional programs in AR^+^ prostate cancer. **(25)** had sub‐nanomolar (DC_50_ < 1 nM) BRD4 degrading activity and a D_max of greater than 99% in AR+ prostate cancer cell lines. It potently suppressed proliferation in VCaP, LNCaP, and 22Rv1 cells with IC_50_ values of 0.016, 0.021, and 0.053 μM, respectively, but was less active in AR‐null lines (PC‐3 and DU145). Mechanistically, **(25)** caused a reduction of BRD4 via UPS degradation, decreased protein levels of c‐Myc, AR, and PSA, and inhibited transcription of AR‐regulated genes (TMPRSS2, ERG, FKBP5). It also induced G0/G1 cell cycle arrest and caspase‐dependent apoptosis of 22Rv1 cells. **(25)** is a potent and selective degrader of BRD4 with a low hook effect and effectively induces BRD4 degradation, making it an attractive candidate for CRPC therapy (Hu et al. 2022).

Sun et al. described compound **(26)**, a first‐in‐class MELK‐degrading PROTAC compound developed through a modular strategy that connects a MELK ligand to pomalidomide via a triethylene glycol linker. Propargyl bromide substitution on intermediate, followed by a click reaction with the E3 ligase ligand byproduct B1, yielded the final compound (**Entry 17,** Table 2). This copper‐free click chemistry approach ensures rapid conjugation and the functional group‐friendliness of this coupling, making it suitable for large‐scale manufacturing. SAR studies further substantiated that the triethylene glycol linker was far superior to the aliphatic ones in facilitating the formation of the ternary complex (MELK‐PLK1) and the degradation of MELK. The identity, purity, structural robustness, and synthetic tractability of **26** were confirmed by analytical methods, including NMR and LC‐MS (Sun et al. 2024).

MELK downregulation is crucial for the **26**‐mediated potent anticancer effect on RAMOS cells. It induces G2/M cell cycle arrest and strong apoptosis through MELK degradation, while suppressing phosphorylation in the mTOR downstream pathway. In vitro, **26** inhibited RAMOS cells with an IC_50_ of 1.8 μM, which is superior to that of classical MELK inhibitors JNJ‐47117096 (IC_50_ = 9.1 μM) and MELK‐8a (IC_50_ = 3.4 μM). Furthermore, **26** showed a higher therapeutic window, as it exhibited little cytotoxicity against MDA‐MB‐231 nontarget cells (CC50 = 13.8 μM) compared with MELK‐8a (CC_50_ = 2.3 μM) and JNJ‐47117096 (CC_50_ = 7.9 μM). Taken together, these results suggest that MELK protein degradation by PROTACs may be a safer approach for Burkitt lymphoma therapy and a possibility for specificity. Overall, **26** addresses the limitation of kinase inhibitor monotherapy and provides a targeted degrader with higher safety and efficacy for B‐cell malignancies induced by MELK overexpression (Sun et al. 2024).

Liu et al. reported that VHL‐recruiting PROTAC compound **(27)** was synthesized by conjugation of a meisoindigo‐derived warhead with a VH032 ligand through a 13‐atom alkyl chain using Cu(I)‐catalyzed azide–alkyne cycloaddition (Click chemistry) (**Entry 18,** Table 2). The synthesis route initiates with the N1‐alkylation of isatin, followed by a Claisen condensation to provide the meisoindigo core, and a CuAAC reaction with an azide‐functionalized VH032 derivative. This linker length is optimal for generating a ternary complex and promoting proteasome degradation. The resulting PROTAC structure retains the same essential interactions while enhancing proteolytic engagement with the VHL E3 ligase to yield the final compound **(27)** in modest yield and good purity, underscoring the modularity and synthetic accessibility to fine‐tune biological activity (Liu et al. 2024).

PROTAC **27** selectively degrades the kinase ataxia telangiectasia mutated (ATM) in colorectal cancer cells through the ubiquitin‐proteasome pathway, resulting in cell cycle arrest and apoptosis. It showed significant cytotoxicity against several cancer cell lines, particularly against SW480 (IC_50_ = 2.59 ± 0.12 μM), SW620 (IC_50_ = 3.70 ± 0.12 μM), and K562 (IC_50_ = 4.06 ± 0.19 μM) cells, but had weak cytotoxicity against normal HIEC‐6 cells (IC_50_ = 23.93 ± 0.39 μM). Investigation of selective degradation and binding of ATM by Western blot and SPR assay results in selective degradation and binding (KD = 1.17 nM) of ATM without inhibiting its kinase activity (IC_50_ > 9.9 μM). Moreover, PROTAC **27** acted synergistically with the ATR inhibitor AZD6738, resulting in robust synthetic lethality and γ‐H2AX accumulation both in vitro and in vivo, thereby highlighting its therapeutic potential for DDR‐deficient tumors (Liu et al. 2024).

Table 2 summarizes the kinase inhibition profiles of the PROTACs, along with their chemical structures and anticancer activities.

## 12 Challenges and Limitations

PROTAC‐mediated targeted protein degradation has significant potential; however, numerous biological and technical barriers limit its wide clinical application and therapeutic efficacy (Hu and Crews 2022). These limitations can occur in a wide variety of areas, ranging from mechanisms of resistance and tissue selectivity to intracellular kinetics, drug‐like properties, and delivery (Chen et al. 2022). To fully realize the potential of PROTACs in precision oncology and other diseases, these obstacles need to be addressed (Mahajan et al. 2025), as summarized in Table 3.

**Table 3:** Clinical challenges of PROTACs.

Challenge	Impact	Mitigation strategies	References
Oral bioavailability	Low absorption due to high molecular weight and polarity	Lipid nanoparticles, prodrug designs	Syahputra et al. (2025); Pike et al. (2020); Li, Guo et al. (2025); Srivastava et al. (2025); Schade et al. (2024)
E3 ligase toxicity	On‐target toxicity in normal tissues (e.g., CRBN in muscle)	Tissue‐specific E3 ligases (RNF114, FEM1B)	Kannt and Đikić (2021); Liang et al. (2024); Moreau et al. (2020); Sobierajski et al. (2024); Mi et al. (2023)
Hook effect	Reduced degradation efficiency at high concentrations	Cooperativity optimization, dose titration	Chen et al. (2022); Riching et al. (2022); Madan et al. (2022); Zhang, Hou et al. (2023); Spitz et al. (2025)
Tumor heterogeneity	Variable degradation in PhosphoTACs due to kinase activity differences	Biomarker‐guided patient stratification	Wang, Zhang, Chen et al. (2024); Mahajan et al. (2025); Yu et al. (2021); Martín‐Acosta and Xiao (2021); Guenette et al. (2022)

Pharmacokinetic limitations remain a significant challenge for PROTAC development (Pike et al. 2020). Due to their large molecular size, polar surface area, and high flexibility, PROTACs often violate the rule of five, which is a guideline established by Lipinski, governing most traditional small molecules (Price et al. 2024). They contribute to a low systemic exposure by enhancing rapid elimination, low membrane permeability, and low oral bioavailability (Bhole et al. 2025). Although a handful of PROTACs have been given orally in preclinical studies, efforts remain to be directed at improving their oral absorption and distribution (Liu, Hu et al. 2022).

Tissue distribution and E3‐ligase selectivity represent other major problems (Hu and Crews 2022). Structure–activity relationships (SARs) for current PROTACs are heavily reliant on just a small fraction of the approximately 600 E3 ligases found in human cells, for example, CRBN, VHL, MDM2, and IAP (Diehl and Ciulli 2022). However, as a function of the cellular context, the degradation effect of these ligases differs, as they are not ubiquitously expressed in all tissues (Kannt and Đikić 2021). In addition, the inappropriate use of particular E3 ligases can lead to knockdown or mutations in the ligase itself, resulting in on‐target off‐tumor toxicity or resistance (Burke et al. 2022).

The cellular localization of the target protein and the E3 ligase also adds another layer of complexity to the phenomenon. Additionally, the POI and the exogenously recruited E3 ligase must co‐localize in the same types of cells for degradation to occur effectively (Wang et al. 2023). For instance, targeting a nuclear protein with a cytosolic E3 ligase (or vice versa) might lead to the insufficient generation and destruction of the ternary complex (Békés et al. 2022). Ligase localization compatibility is thus an essential design parameter, particularly when targeting a compartment‐specific or noncanonical protein (Scott et al. 2024).

Resistance to PROTACs is now being identified and mimics traditional treatment resistance (Song et al. 2024). These mechanisms include alterations in the expression or mutation of the reconstituted E3 ligase, changes in the binding site of the target protein, and an increase in the level of deubiquitinases (DUBs) that remove ubiquitin chains before their recognition by proteasomes (Qadir et al. 2024). Moreover, PROTAC‐refractory clones unable to degrade efficiently could emerge in response to evolutionary pressure from long‐term PROTAC treatment (Mahajan et al. 2025).

TF‐PROTACs face significant delivery challenges because they often utilize DNA or RNA oligonucleotides as binding ligands (Zhang et al. 2025). Their poor endosomal escape, nuclease degradation, and low stability necessitate the use of more advanced delivery systems, such as lipid nanoparticles or conjugation to cell‐penetrating peptides (Wang et al. 2023). Numerous next‐generation PROTACs do not achieve intracellular target validation at therapeutically relevant concentrations without the presence of efficient delivery methods (Wang, Zhang, Chen et al. 2024).

Target selection is a limitation. Although PROTACs can degrade a wide range of proteins, numerous constraints make their application challenging, including the need for finding ligands that would bind with high affinity to the target/the E3 ubiquitin ligase, the involvement of lysine residues in ubiquitination, and the requirement of keeping the protein in a conformation that allows the formation of a ternary complex (Castellani et al. 2023). Even with the progress made with next‐generation agents, targeting specific proteins, including those with disordered or structureless domains (e.g., TFs), remains especially challenging because they can be physically flat, structurally disorganized, or functionally diverse (Troup et al. 2020).

Ultimately, while PROTACs represent a groundbreaking therapeutic modality, numerous biological, pharmacological, and translational challenges remain to be addressed (Wang, Zhang, Yu et al. 2024). To further breakthrough these barriers and bring PROTACs into broader clinical use, the development of novel E3 ligases, improved delivery strategies, and rational degrader design will be necessary in the future (Liu, Hu et al. 2022).

## 13 Clinical and Preclinical Landscape

The transition of PROTACs from concept to clinical candidate is a crucial step in the development of new drugs (Nieto‐Jiménez et al. 2022). Notably, targeted degradation of essential drivers of cancer disease, such as kinases, transcriptional factors, and hormone receptors, has been reported for PROTACs, initially verified in preclinical model systems (Liang et al. 2024). These early successes have led to clinical translation, and there are now several PROTACs in human clinical trials, primarily being tested for the treatment of hematologic and hormone‐dependent cancers (Liu, Hu et al. 2022).

Two clinical leads, in particular, have garnered attention: Arvinas' ARV‐110 and ARV‐471. In metastatic castration‐resistant prostate cancer (mCRPC), ARV‐110 is an orally bioavailable PROTAC that degrades the AR (Han and Sun 2022). In Phase I/II trials, it showed a promising PSA reduction and target engagement in a subset of patients (Dubey et al. 2024). In contrast, ARV‐471, which is being developed in collaboration with Pfizer, focuses on the ER alpha (ERα) in ER‐positive, HER2‐negative breast cancer (Hamilton et al. 2025). Clinical trials are currently in late‐stage development with reasonable tolerability and signs of efficacy (Guedeney et al. 2023). Preclinical data showed potent ER degradation and tumor regression in xenograft models (Xie et al. 2023), as profiled in Table 4.

**Table 4:** Clinical‐stage PROTACs and key challenges.

PROTAC	Target	Indication	Key limitation	References
Vepdegestrant (ARV‐471)	Estrogen receptor (ER)	Breast cancer	Muscle waste (CRBN‐mediated)	Hamilton et al. (2025); Rej et al. (2023); Min et al. (2024); Hamilton et al. (2024); Avery et al. (2024)
Bavdegalutamide (ARV‐110)	Androgen receptor (AR)	Prostate cancer	Limited efficacy in AR‐LBD mutants	Snyder et al. (2025); Dubey et al. (2024); Movahed et al. (2024); Ha et al. (2022); Israel et al. (2024)
DT2216	BCL‐XL	Leukemia	Thrombocytopenia	Zhang et al. (2019); Jaiswal et al. (2023); He et al. (2020)
CFT7455	IKZF1/3	Myeloma	Neutropenia	Li and Crews (2022); Berdeja et al. (2021); Thomenius et al. (2023); Kong and Jones (2023)
MDM2	E3 ubiquitin ligase and p53	Breast and lung cancers	Amplification of the MDM2 gene locus (12q15)	Han et al. (2022); Goerg et al. (2025); He et al. (2021); Li, Cai et al. (2024); Vicente and Salvador (2022)

Several classes of PROTACs targeting oncoproteins, such as EGFR, BTK, BCL‐XL, BRD4, CDK6, or STAT3, have been designed at the preclinical level (Liu, Hu et al. 2022; Nieto‐Jiménez et al. 2022; Vicente and Salvador 2024). For instance, the BRD4‐targeting PROTACs, such as dBET1 and ARV‐825, selectively deplete BRD4 and MYC‐driven transcription in hematologic malignancies (Wu et al. 2021). Likewise, through the total degradation of proteins and the resistance mechanisms involving kinase reactivation or bypass signaling, CDK6 degraders are undoubtedly superior to traditional CDK4/6 inhibitors (Kumarasamy et al. 2023).

In particular, dual‐target PROTACs, such as DP‐V‐4 (co‐degrading PARP and EGFR), have shown potential in combating adaptive resistance in NSCLC, where both single‐target and multi‐target resistance mechanisms are present in the same patient tumors (Cordani et al. 2024). These bifunctional degraders enhance the efficacy of drugs in monotherapy‐resistant models by mitigating pathway redundancy (Cao et al. 2022).

Additionally, preclinical efficacy against previously undruggable targets has been demonstrated by new PROTAC technologies such as TF‐PROTACs and PhosphoTACs (Kubryń et al. 2025). While PhosphoTACs utilize phosphorylation‐dependent protein‐protein interactions to degrade hyperactivated signaling molecules, such as FRS2α, selectively, TF‐PROTACs employ DNA oligonucleotides to regulate the degradation of TFs like STAT3, which lack specific binding sites (Liu, Chen, Kaniskan et al. 2021; Bond and Crews 2021). Although these approaches are still in the preclinical stage, they represent a significant advancement in precise targeting based on the protein's functional class or activation status (Li, Song et al. 2022).

The applications of PROTACs are rapidly expanding into other disease areas, including neurodegeneration, inflammation, and infectious diseases, despite most clinical efforts having been focused on oncology (Zhu et al. 2025). For example, Tau‐, α‐Synuclein‐, or mutant Huntingtin‐specific PROTAC compounds have been developed for Alzheimer's and Parkinson's diseases; however, these PROTACs have not been tested in clinical studies to date (Kong et al. 2025).

The broad applicability of the strategies (utilized in therapy‐oncology patients) and their selective mode of action, which overcomes resistance, is emphasized by the clinical and preclinical landscape of PROTACs (Wang, Zhang, Yu et al. 2024). The successful ongoing clinical candidates confirm the platform, but they also highlight the necessity of translational biomarkers, ligand optimizations, and an innovative design towards sustainable success (Wang, Zhang, Chen et al. 2024). The therapeutic landscape of numerous human diseases could be transformed with the development of new generations of PROTACs integrated into personalized medicine (Bhole et al. 2025).

## 14 Future Perspectives and Opportunities

The success of PROTACs is a harbinger of a new era in drug development, specifically the targeting of protein degradation (TPD) (Wang, Zhang, Chen et al. 2024). Future efforts will likely overcome the current limitations imposed on PROTAC's chemical space, targetable proteome, and therapeutic window (Scott et al. 2024). New therapeutic degraders will need to rely on innovative chemistries, exciting delivery systems, and novel biology to overcome technological challenges and target unmet patient needs (Liang et al. 2024).

The diversification of E3 ligase is a significant improvement (Hall et al. 2024). The limited number of E3 ligases, such as CRBN and VHL, that are targeted by most current PROTACs may render tissue selectivity challenging or lead to resistance (Kamaraj et al. 2024). Discovery and application of new E3 ligases with tissue‐ or disease‐specific expression will be a key part of future work (Sobierajski et al. 2024). The clinical success of ARV‐110 and ARV‐471 has enabled the therapeutic validation of PROTACs, sparking widespread interest in their clinical use in oncology and other indications. This success has also led to the recruitment of understudied ligases, such as KEAP1 (Dubey et al. 2024). As the science matures, it appears that precision degrader‐based therapies, which can be tailored to the patient's individual disease drivers, may become increasingly common (Kanbar et al. 2024). With sustained interdisciplinary innovation, PROTACs are well‐positioned to become a central component of the next‐level therapies that transform the way we remove unhealthy proteins and treat complex, resistant diseases (Zhu et al. 2025). In addition to developing derivatives of optimal natural binders or mutants that bind the target with greater affinity and stability, or to target alternative E3 ligases such as F4, MDMX, or FBW7, attempts such as ligand screening, covalent targeting, and molecular glues may result in better degradability with lower off‐target toxicity (Sosič et al. 2022; Troup et al. 2020).

The interest in expanding PROTACs to other target proteins and new ligases is growing (Berkley et al. 2025). Through advances such as TF‐PROTACs, PhosphoTACs, and oligonucleotide‐directed degraders, proteins that were previously considered undruggable, including TFs, scaffolding proteins, RNA‐binding proteins, and protein aggregates with misfolding, are now within reach (Kubryń et al. 2025). Additionally, conditional degraders offer strong spatiotemporal control through various stimuli, including light, pH, and hypoxia. This might leave room for tissue‐selective or on‐demand degradation (He et al. 2025).

Opportunities outside of cancer indications are available. Although the emphasis has been on oncology, PROTACs are increasingly becoming a subject of study for immune and viral afflictions, as well as for neurological conditions such as Alzheimer's, Parkinson's, and Huntington's (Kong et al. 2025). For example, preclinical work suggests the potential of PROTACs targeting tau, mutant huntingtin, or viral proteins such as HBx (from hepatitis B) (Kubryń et al. 2025). The potential to degrade these pathogenic but non‐enzyme proteins is a potential new treatment for diseases once thought to be incurable (Zhu et al. 2025).

Another aspect that has yet to be investigated in the future is the establishment of oral, CNS‐permeable, and cell type‐specific degraders (Cecchini et al. 2021). However, these molecules are big and polar; the development of brain‐penetrable (blood‐brain barrier, BBB) PROTACs is challenging (Syahputra et al. 2025). The bioavailability and CNS penetration of PROTACs could potentially be improved by employing more advanced medicinal chemistry strategies, including intramolecular hydrogen bonding, macrocyclization, and prodrug approaches (Liu, Kalogeropulou et al. 2022).

Furthermore, it is also expected that combinational therapies with PROTACs and other compounds, including kinase inhibitors, immune checkpoint inhibitors, and DNA damage response (DDR) modulators, as well as others, will enhance efficacy and prevent resistance (Wang, Zhang, Chen et al. 2024). For example, in triple‐negative breast cancer models, a PARP inhibitor combined with a BRD4 PROTAC displayed potent antitumor synergistic benefits (Dogheim and Amralla 2023).

The future identification of degraders is also likely to be expedited by advances in proteomics, CRISPR‐based screening, and AI modeling (Gharbi and Mercado 2024). Degradable hotspots on proteins can be identified by degron mapping approaches and high‐throughput screens for degraders (Ou et al. 2025). AI‐predicted ternary complex formation and degradation probabilities should remove much of the guesswork from the equation and compensate for logical design (Danishuddin et al. 2023). Finally, the field is moving toward individualized degraders, with patients' mutations, splice variants, or expression profiles used to direct the choice of what to target with degradation and the choice of a ligase (Bhole et al. 2025). This will, in particular, introduce PROTACs into the world of precision medicine for cancers with well‐characterized molecular markers or known mechanisms of resistance (Rutherford and McManus 2024).

The possibilities of PROTACs are so much more than just the mere degradation of proteins (Békés et al. 2022). This comprises a platform technology that has the potential to impact therapeutic paradigms across specialties by reprogramming the proteome (Wang, Zhang, Chen et al. 2024). With new advances in biology, chemistry, and delivery technology, PROTACs are expected to become a vital part of medicine in the 21st century (Lin et al. 2025).

## 15 Conclusion

By facilitating the selective breakdown of disease‐causing proteins, including those previously considered undruggable, PROTACs have significantly altered the therapeutic landscape. They provide an alternative to the conventional occupancy‐driven inhibition paradigm by hijacking the endogenous UPS and replacing it with a catalytic, event‐driven system. They have established themselves as potent instruments to overcome resistance and enhance therapeutic precision due to their modularity, catalytic mode of action, and possibility for lower dosage. Several next‐generation PROTAC platforms that have increased the breadth and accuracy of this method were highlighted in this review. These include TF‐PROTACs, which use oligonucleotide‐guided recruitment of TFs; dual‐target degraders (like DP‐V‐4) that can target compensatory signaling pathways; and phosphoTACs, which introduce activation‐state selectivity by breaking down hyperphosphorylated proteins. Furthermore, new tools such as PhosTACs, photocaged PROTACs, and folate‐caged PROTACs offer additional layers of cell‐type selectivity and spatiotemporal control, thereby improving safety and efficacy. The lack of tissue‐specific E3 ligases, poor oral bioavailability, restricted cell permeability, and variability in ternary complex formation remain some of the limitations, despite these developments representing a considerable advancement. To overcome these challenges, an interdisciplinary approach spanning high‐throughput proteomics, medicinal chemistry, structural biology, and AI‐driven modeling will be necessary to rationalize degrader design and more accurately predict degradation kinetics. Notably, clinical‐stage compounds such as ARV‐110 and ARV‐471 have confirmed the therapeutic value of PROTACs, providing opportunities for new applications in oncology and potentially other fields. The development of precision degrader medicines that can adapt to the unique characteristics of each patient and intricate disease networks appears to be progressing as research advances. With further development, PROTACs have the potential to completely transform therapeutic protein targeting, enhancing current therapies.

## Conflicts of Interest

The authors declare no conflicts of interest.

## Data Availability

This manuscript does not involve any experimental work.
